# Urine sampling techniques in symptomatic primary-care patients: a diagnostic accuracy review

**DOI:** 10.1186/s12875-016-0465-4

**Published:** 2016-06-08

**Authors:** Anne Holm, Rune Aabenhus

**Affiliations:** Research Unit for General Practice and Section of General Practice, Department of Public Health, University of Copenhagen, Øster Farimagsgade 5, PO box 2099, 1014 Copenhagen, Denmark

**Keywords:** “Urinary tract infections” [Mesh], “Urine” [Mesh], “Specimen handling” [Mesh], “Urine specimen collection” [Mesh], “Primary health care” [Mesh], “General practice” [Mesh]

## Abstract

**Background:**

Choice of urine sampling technique in urinary tract infection may impact diagnostic accuracy and thus lead to possible over- or undertreatment. Currently no evidencebased consensus exists regarding correct sampling technique of urine from women with symptoms of urinary tract infection in primary care. The aim of this study was to determine the accuracy of urine culture from different sampling-techniques in symptomatic non-pregnant women in primary care.

**Methods:**

A systematic review was conducted by searching Medline and Embase for clinical studies conducted in primary care using a randomized or paired design to compare the result of urine culture obtained with two or more collection techniques in adult, female, non-pregnant patients with symptoms of urinary tract infection. We evaluated quality of the studies and compared accuracy based on dichotomized outcomes.

**Results:**

We included seven studies investigating urine sampling technique in 1062 symptomatic patients in primary care. Mid-stream-clean-catch had a positive predictive value of 0.79 to 0.95 and a negative predictive value close to 1 compared to sterile techniques. Two randomized controlled trials found no difference in infection rate between mid-stream-clean-catch, mid-stream-urine and random samples.

**Conclusions:**

At present, no evidence suggests that sampling technique affects the accuracy of the microbiological diagnosis in non-pregnant women with symptoms of urinary tract infection in primary care. However, the evidence presented is in-direct and the difference between mid-stream-clean-catch, mid-stream-urine and random samples remains to be investigated in a paired design to verify the present findings.

**Electronic supplementary material:**

The online version of this article (doi:10.1186/s12875-016-0465-4) contains supplementary material, which is available to authorized users.

## Background

Symptomatic urinary tract infection (UTI) in women is a common condition in general practice, and every day general practitioners or their staff instruct women in delivering urine samples for examination [1]. The main concern when sampling urine is that inadequate handling may increase the risk of contamination in turn leading to overdiagnosis and overtreatment of UTI. Sterile collection of urine samples can be performed using suprapubic puncture or urethral catheterization and use of these collection techniques could possibly reduce contamination and thereby overdiagnosis and overtreatment. However, in a primary care setting these methods are considered obsolete today due to the associated discomfort for the patient and a minor risk of iatrogenic infection and other complications. Current methods include i) mid-stream-clean-catch technique (MSCC) where the patient is instructed to clean the labia before voiding using tap water, soap or disinfectants, ii) mid-stream urine (MSU) without prior cleaning, iii) random samples delivered without instruction or iv) home-voided samples with or without standardized transport media. These sampling techniques are mostly based on tradition or expert opinion and ease-of-use for patient and doctor rather than stringent evidence. A study from 2000 conducted in primary care found no evidence that sampling technique affected contamination rate or infection rate in urine samples [2], but new evidence within this area is often questioned and debated [3–5]. Since sampling techniques (MSCC, MSU, random samples and home voiding) differ extensively in preparation time and discomfort, ease-of-use for doctors as well as their patients, it is relevant to review their diagnostic yield. The aim of this study was to conduct a systematic review to determine the accuracy of urine culture from different sampling techniques in symptomatic patients in primary care.

## Method

### Literature search

We searched Medline and Embase for clinical studies conducted in primary care published before May 2015 in English, Swedish, Danish or Norwegian. Combinations of the words “urinary tract infection”, cystitis, bacteriuria, urine, specimen, handling, urinalysis, collection, midstream and” clean catch” were used. To identify more studies from before 1970, a slightly different search-string was used for the older studies in Medline. The literature search and inclusion of studies was performed by AH. The full search strings can be seen in Appendix A.

#### Inclusion criteria

Clinical studies randomizing or using a paired design to compare the result of urine culture obtained with two or more collection techniques in adult, self-helped, non-pregnant (and not post-partum) women with symptoms of UTI in primary care (general practice, outpatients clinics or comparable settings). We did not discriminate between complicated and uncomplicated cases of UTI.

#### Exclusion criteria

- Studies investigating mainly patients who were not self-helped, were asymptomatic, pregnant, children or men (wrong group)
- Studies conducted in the secondary sector (wrong setting)
- Studies using other modalities than culture as reference (wrong gold standard)
- Studies where data for the selected outcome was not available (missing data)
- Studies using a different design than described in the inclusion criteria (wrong design)

The references of included studies were screened and experts in the field were contacted to provide additional literature.

### Data extraction

Data from included studies were entered into a data-form with information on setting, number of patients, age, inclusion- and exclusion-criteria for the study, reference and index text, the assigned cut off for infection vs. contamination, the bacteria identified and study design. Data on absolute numbers of infected urine samples, true and false positives and negatives or predictive values of one sampling method versus another were likewise extracted from the included studies. If these measures were not directly provided in the article, we calculated them if possible. Selected outcomes were dichotomized for the planned analyses as negative/positive culture. Culture results presented as equivocal and contaminated were grouped with the negative results. Data from the relevant patients were extracted when studies also included patients covered by the exclusion criteria. Data extraction was done by both authors and discrepancies were discussed and corrected. When data was not available or incomplete we referred from contacting authors, as most studies were more than 10 years old.

### Definition of reference standard

Assuming an increasing contamination rate in the order of: 1) Suprapubic puncture, 2) urethral catheterization samples, 3) MSCC, 4) MSU, 5) Random samples, 6) Home-voided urine, the least contaminated was used as reference and the most contaminated as index test. For example, if a study investigated both MSCC and random urine sampling in a paired design, MSCC was used as reference standard and random samples as index test.

### Study designs

This review included both paired studies and randomized controlled trials (RCT). RCTs were analysed separately.

### Quality assessment

The included studies were evaluated using QUADAS-2 for assessment of diagnostic accuracy studies [6]. No study was excluded based on low quality according to this tool. Both studies using paired samples and randomized controlled trials were assessed with QUADAS-2.

### Data analysis

The specified dichotomized outcomes were used to calculate predictive values, sensitivity and specificity in paired studies. The generated sensitivity and specificity values were used to create forest plots on the diagnostic accuracy. Diagnostic accuracy plots were performed using Review Manager (RevMan) Version 5.3. Copenhagen: The Nordic Cochrane Centre, the Cochrane Collaboration, 2014.

## Results

### Literature search

**Fig. 1:**
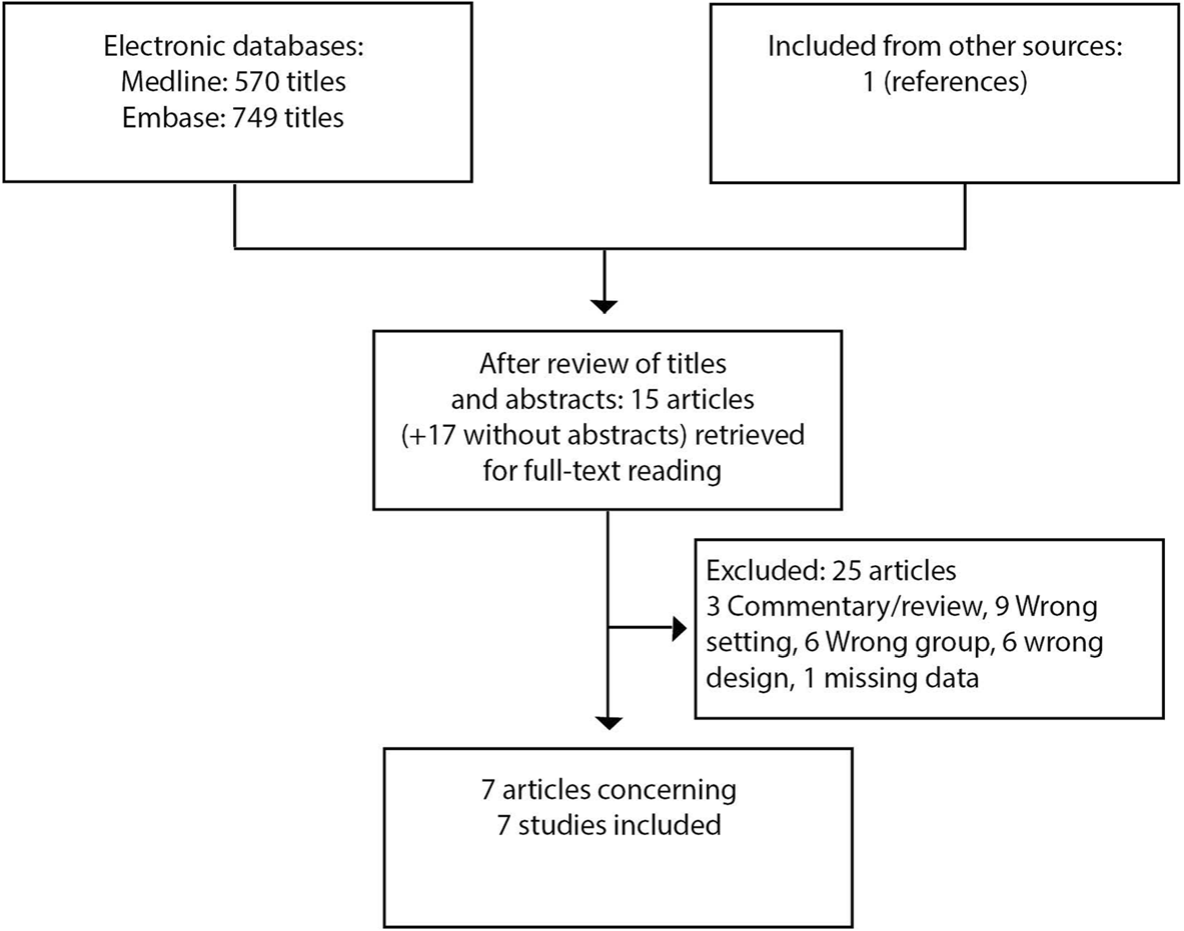
Short legend: data collection

The initial search resulted in 570 titles in Medline and 749 titles in Embase. After review of titles, abstracts and articles we included seven full text articles presenting results from seven studies investigating urine sampling technique in 1062 non-pregnant women with symptoms of UTI in primary care. A flow diagram of the literature search and review of titles, abstracts, and articles is shown in Fig. 1. Two of the studies were from general practice while the remaining five were from outpatient clinics or student clinics. The included studies are shown in Table 1. A list of excluded studies is provided in Appendix B.

**Table 1:** Characteristics of included studies

Study	Setting	Design	Patients (n)	Incidence	Technique	Cutoff index^a^	Cutoff reference^a^
Hooton 2013	Outpatient clinic	Paired samples	202^b^	0.70	MSCC vs. Cat	≥10 cfu/ml	≥10 cfu/ml
Lifshitz 2000	University clinic	RCT	242	0.55	Random vs. MSCC^c^	≥ 10^2^ cfu/ml	≥ 10^2^ cfu/ml
Baerheim 1990	General practice	Paired samples	73	0.74	Home vs. MSCC	≥ 10^4^ cfu/ml	≥ 10^4^ cfu/ml
Walter 1989	Outpatient clinic	Paired samples	105	0.40	MSCC vs. Cat	≥ 10^5^ cfu/ml	≥ 10^5^ cfu/ml
Bradbury 1988	General practice	RCT	158	0.25	MSU vs. MSCC	> 10^5^ cfu/ml	> 10^5^ cfu/ml
Stamm 1982	Outpatient clinic/student clinic	Paired samples	187	0.52	MSCC vs. Cat/Sup	Reporting absolute counts	≥10 cfu/ml
Mabeck 1969	Outpatient clinic	Paired samples	95	-	MSCC vs. Sup	Reporting absolute counts	Reporting absolute numbers

Detailed legend: Characteristics of included studies. ^a^The definition has been simplified. ^b^Reporting the number of samples not patients. ^c^MSCC and MSCC + vaginal tampoon. *RCT* Randomized controlled trial; *MSCC* Mid-stream-clean-catch; *MSU* Mid-stream-urine; *Cat* Urethral Catheterization; *SUP* suprapubic puncture; *cfu* colony-forming units

### Quality of included studies according to Quadas-2

The quality of the included studies is summarized in Table 2. Generally the studies were judged to be of moderate to high risk of bias. No study was considered having low risk of bias. The most common error was lack of blinding of the interpreter to the results of the index and reference tests or lack of reporting of blinding. The applicability of the studies was not regarded a general concern. The full quality assessment is described in Additional file 1.

**Table 2:**
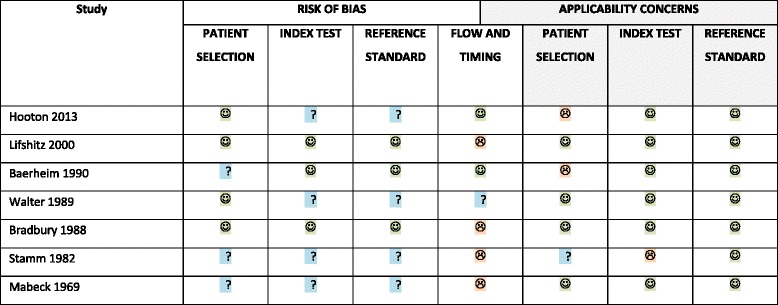
Quality of included studies assessed using Qaudas-2

Detailed legend:  Low Risk  High Risk  Unclear Risk

### Data from included studies

#### Paired design studies

Four studies used a paired design to compare MSCC urine samples to samples obtained with urethral catheterization or suprapubic puncture (*n* = 589) [7–10]. Urethral catheterization and suprapubic puncture are essentially sterile techniques and served as reference e.g. gold standard. Two of these studies applied ≥10 cfu/ml as the cut-off for infection in both index- and reference-test, one study used a cut-off of ≥ 10^5^ cfu/ml and one reported absolute counts for both index- and reference-test (Table 1). The positive predictive value (PPV) of a MSCC sample varied according to the chosen cut-off for infection: cutoff: ≥10 cfu/ml 0.79 (0.71-0.86); cutoff: ≥ 10^5^ cfu/ml 0.95 (0.83-0.99). The negative predictive value of a MSCC was close to 1 in all four studies. The accuracy found in the four studies is shown in Table 3. The achieved specificity was influenced by the selected cut-off levels, with higher thresholds corresponding to increasing specificity. We did not perform a meta-analysis or calculate heterogeneity as the applied cut-offs varied considerable thus impeding a meaningful pooling of the results.

**Table 3:**
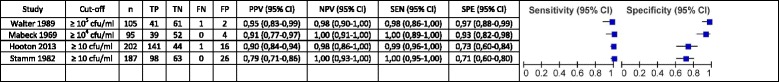
MCSS vs. sterile samples

Detailed legend: Diagnostic accuracy of mid-stream-clean-catch samples vs. urethral catheterization/suprapubic puncture. 95 % confidence intervals in brackets. A cut-off of ≥ 10^4^ cfu/ml has been chosen in the study by Mabeck. *TP* True positives; *TN* True negatives; *FN* False negatives; *FP* False positives; *PPV* Positive predictive value; *NPV* Negative predictive value; *SEN* Sensitivity; *SPE* Specificity

One study investigated home-voided samples against MSCC taken in general practice [11]. This study found a high PPV of home-voided samples of 0.92 (0.81-0.98), but a lower NPV of 0.71 (0.48-0.88). The results of this study are shown in Table 4.

**Table 4:**
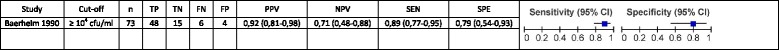
Home-voided samples vs. MSCC

Detailed legend: Diagnostic accuracy of home-voided samples vs. mid-stream-clean-catch samples. 95 % confidence intervals in brackets. *TP* True positives; *TN* True negatives; *FN* False negatives; *FP* False positives; *PPV* Positive predictive value; *NPV* Negative predictive valuxe; *SEN* Sensitivity; *SPE* Specificity

The studies by Stamm and Mabeck reported absolute counts of colony-forming units in the voided urine samples and this allowed us to investigate the current cut-off for primary uropathogens of 10^3^ cfu/ml in voided urine samples against 10 cfu/ml in suprapubic puncture [12]. Using these current cut-offs we calculated the sensitivity of MSCC to be 0.81 (0.71-0.88) in the study by Stamm and 0.96 (0.85-0.99) in the study by Mabeck. Corresponding specificities were 0.90 (0.82-0.95) in the study by Stamm and 0.59 (0.43-0.73) in the study by Mabeck.

### Randomized controlled trials

Two randomized controlled trials were identified comparing MSU or random samples to MSCC with infection rate and contamination rate in the randomization-groups as their primary outcomes (number of patients = 400) [2, 13]. Because of the randomized design, accuracy could not be calculated from these studies. The studies are shown in Table 5. None of the studies found significant differences in infection rate or contamination rate between sampling techniques.

**Table 5:** Randomized controlled trials and infection rates

Study	Technique	Patients (n)	Incidence	Index infected (95 % CI)	Reference infected (95 % CI)
Bradbury 1988	MSU vs. MSCC	158	0.25 (0.18-0.31)	0.25 (0.14-0.35)	0.25 (0.16-0.34)
Lifshitz 2000	Random vs. MSCC	242	0.55 (49–61)	0.57 (0.46-0.68)	0.53 (0.45-0.61)

Detailed legend: Infection rates in randomized controlled trials included in the review. 95 % confidence intervals in brackets. *MSCC* mid-stream clean catch; *MSU* Mid-stream urine; *Random* random sample without instruction

## Discussion

This diagnostic accuracy review is the first to assess the available evidence from different urine sampling techniques on symptomatic patients with suspected UTI in primary care. Overall, we did not find consistent evidence to suggest important differences in diagnostic accuracy among the included urine sampling techniques (MSCC, MSU or random voiding). The slightly lower specificity of voided samples compared to invasive sampling techniques (suprapubic puncture and catheter) will cause 5–10 % of healthy patients to be overdiagnosed. This does not, in our opinion, outweigh the discomfort and risk of complications associated with sterile techniques. The quality of the studies was moderate and substantial heterogeneity was present between study designs and applied diagnostic cut-offs. With the available evidence, each general practitioner can choose freely the sampling technique most appropriate for his or her practice and patients.

The current review included two studies from general practice and 5 from outpatient clinics or student clinics. Participants were symptomatic patients under investigation for urinary tract infection. We have no reason to suspect the included patients differ from the average UTI patient in primary care. Thus we believe the results can be considered applicable to most primary care settings including general practice.

The included methods of urine sampling included, the different cut-offs for infection applied and the time span between studies of up to 50 years does however suggest that the overall results regarding their diagnostic accuracy should be considered with caution.

The current consensus regarding a cut-off for infection (*eg.* 10^3^ cfu/ml for primary uropathogens) was not directly assessed in any of the studies, but we calculated the sensitivity and specificity based on the two studies by Mabeck and Stamm. While the sensitivity was above 0.80 in both studies, the specificity differed between studies and was low (0.59) in the study by Mabeck. However, this could be a chance finding and caution should be excised when interpreting these results as they are based on few older studies and we do not know if this result would still apply today with current microbiological procedures. Furthermore, current cut-offs are based on microbiological assessments and have, to our knowledge, never been validated in relation to patient-relevant outcomes like cure-rate or impact on daily activities. The development of such patient-centred outcomes may be more applicable to a general practice setting.

The European urine analysis guideline recommends a MSCC without detergents [12]. However, this guideline is based on studies including pregnant, asymptomatic as well as hospitalized patients and their conclusions do not necessarily apply to the average patient in general practice. Studies based in secondary care have found varying accuracy of voided urine samples depending on their patient group, design and gold standard [14–18]. However, studies investigating symptomatic, otherwise healthy women seem to essentially reproduce our findings [19, 20].

## Conclusions

The present review does not present evidence to suggest one urine sampling technique over another according to diagnostic performance; rather this should at present depend on ease of use and convenience for patients and practices. This lack of evidence is in part due to few available studies and further testing on current diagnostic cut-offs as well as new patient-centred outcomes is warranted.

## Abbreviations

FN, false negatives; FP, false positives; MSCC, mid-stream-clean-catch technique; MSU, mid-stream urine; NPV, negative predictive value; PPV, positive predictive value; RCT, randomized controlled trials; SEN, sensitivity; SPE, specificity; TN, true negatives; TP, true positives; UTI, urinary tract infection.

## Appendix A

### Search Strings

SEARCH:

Search string Medline 01.01.1971 – 31.05.2015: ("Urinary Tract Infections"[Mesh] OR "Cystitis"Mesh OR “Urinary Tract Infection*" OR Cystitis OR "Bacteriuria"Mesh OR Bacteriuria) AND (Urine OR “Urine”Mesh]) AND ("Specimen Handling"[Mesh] OR "Urinalysis"[Mesh] OR "Urine Specimen Collection"Mesh+ OR Collection OR midstream OR “clean catch”) NOT(“Animals”[Mesh] NOT “Humans”Mesh]) NOT "Pregnancy"Mesh NOT “Child”[Mesh] NOT “Infant”[Mesh] NOT “Male”[Mesh]

Search string Medline 01.01.1955 - 31.12.1970: ("Urinary Tract Infections"[Mesh] OR "Cystitis"[Mesh] OR “Urinary Tract Infection*" OR Cystitis OR "Bacteriuria"[Mesh] OR Bacteriuria) AND (Urine OR “Urine”[Mesh]) AND ("Specimen Handling"[Mesh] OR "Urinalysis"[Mesh] "Urine Specimen Collection"[Mesh] OR Collection OR midstream OR “clean catch” OR “Urine/microbiology”[Mesh]) NOT(“Animals”[Mesh] NOT “Humans”[Mesh]) NOT "Pregnancy"[Mesh] NOT “Child”[Mesh] NOT “Infant”[Mesh] NOT “Male”[Mesh]

Search string Embase: (urinary tract infection/or cystitis.mp. or cystitis/or bacteriuria/or bacteriuria.mp.) And (urine/or urine.mp.) And (collection or specimen or midstream).af. And (woman or women or female).af.

Filter: Language = English, Swedish, Danish, Norwegian

## Appendix B

**Table 6:** Complete list of excluded studies

Title	Author	Year	Identified	Excusion	Excluded after
Abnormal urinalysis results are common, regardless of specimen collection technique, in women without urinary tract infections.	Frazee B.W. Enriquez K. Ng V. Alter H.	2015	Embase	Wrong group	Abstract
A midstream urine collector is not a good alternative to a sterile collection method during the diagnosis of urinary tract infection.	Verliat-Guinaud J, Blanc P, Garnier F, Gajdos V, Guigonis V.	2015	Medline	Wrong group	Abstract
Re: Voided midstream urine culture and acute cystitis in premenopausal women.	Schaeffer E.M.	2014	Embase	Commentary/review	Abstract
Associations between individual lower urinary tract symptoms and bacteriuria in random urine samples in women. V.	Sorrentino F, Cartwright R, Digesu GA, Tolton L, Franklin L, Singh A, Greco P, Khullar V	2014	Medline	Wrong setting	Abstract
Infection: Utility of midstream urine cultures questioned.	Payton S.	2014	Medline	Commentary/review	Abstract
Voided midstream urine culture is a good test for acute cystitis in premenopausal women.	Cox L, Clemens JQ.	2014	Medline	Commentary/review	Article
Easy peezy: A patient satisfaction survey on an innovative device for collection of mid-stream urine (MSU) samples.	Khorsandi M. Hussain B. Chow W.	2013	Embase	Wrong design	Abstract
Peezy at ease: Our initial 106 patients experience on an innovative device for collection of Mid-Stream Urine (MSU) samples.	Chow W.M. Hussain B.	2013	Embase	Wrong design	Abstract
Effect of urogenital cleaning with paper soap on bacterial contamination rate while collecting midstream urine specimens	Shrestha R, Gyawali N, Gurung R, Amatya R, Bhattacharya SK.	2013	Medline	Wrong setting	Article
Urine collection in the emergency department: what really happens in there?	Frazee BW, Frausto K, Cisse B, White DE, Alter H.	2013	Medline	Wrong setting	Abstract
Urine specimen collection: how a multidisciplinary team improved patient outcomes using best practices.	Dolan VJ, Cornish NE.	2013	Medline	Wrong setting	Abstract
“Mixed growth of doubtful significance” is extremely significant in patients with lower urinary tract symptoms.	Sathiananthamoorthy S. Swamy S. Kupelian A. Horsley H. Gill K. Collins L. Malone-Lee J.	2012	Embase	Wrong design	Abstract
The impact of improperly collected urine cultures on patient treatment in the emergency department.	Francis K. Lucente K.M. Kim Y.	2012	Embase	Wrong design	Abstract
Managing UTI in primary care: should we be sending midstream urine samples?	Hay AD.	2010	Medline	Commentary/review	Abstract
A comparative study on bacterial cultures of urine samples obtained by clean-void technique versus urethral catheterization.	Lau AY, Wong SN, Yip KT, Fong KW, Li SP, Que TL.	2007	Medline	Wrong group	Abstract
Comparison of sampling methods for urine cultures.	UnlÃ¼ H, Sardan YC, Ulker S	2007	Medline	Wrong setting	Abstract
Effect of perineal cleansing on contamination rate of mid-stream urine culture.	Blake DR, Doherty LF.	2006	Medline	Wrong group	Abstract
Obtaining a catheter specimen of urine.	Gilbert R.	2006	Medline	Commentary/review	Article
Taking a midstream specimen of urine.	Gilbert R.	2006	Medline	Commentary/review	Abstract
A novel midstream urine-collection device reduces contamination rates in urine cultures amongst women.	Jackson S.R. Dryden M. Gillett P. Kearney P. Weatherall R.	2005	Embase	Wrong group	Abstract
Catheter specimens of urine: an audit of practice.	Gilbert R, Henderson S.	2005	Medline	Wrong design	Abstract
Contamination of urine specimens did not differ with collection technique in women with acute dysuria.	Lifshitz E. Kramer L.	2001	Embase	Commentary/review	Abstract
Collection and transport of urine for culture.	Perera CU.	2001	Medline	Commentary/review	Abstract
Honey jars and diagnosis of urinary tract infections--ascent quality work.	Forsum U.	2001	Medline	Wrong design	Article
A technique for collection of uncontaminated urine for culture from female patients.	Gleason D.M. Bottaccini M.R. Reilly R.J. McNeill J.	2000	Embase	Wrong group	Article
The midstream muddle.	Bannatyne RM.	2000	Medline	Commentary/review	Abstract
Urine collection and culture in elderly people.	Clague J, Horan M.	1998	Medline	Wrong design	Article
A simple and efficient urine sampling method for bacteriological examination in elderly women.	Michielsen WJ, Geurs FJ, Verschraegen GL, Claeys GW, Afschrift MB.	1997	Medline	Wrong group	Article
Assessment of urine collection technique for microbial culture.	Prandoni D, Boone MH, Larson E, Blane CG, Fitzpatrick H.	1996	Medline	Wrong group	Article
Collecting clean-catch urine in the nursing home: obtaining the uncontaminated specimen.	Brazier A.M. Palmer M.H.	1995	Embase	Commentary/review	Abstract
Bacteriuria--sampling methods and significance.	Pfau A.	1994	Medline	Wrong design	Article
Collection of urine for culture.	Jaffe JS.	1994	Medline	Commentary/review	Abstract
Does a clean-catch urine sample reduce bacterial contamination?	Leisure MK, Dudley SM, Donowitz LG.	1993	Medline	Wrong group	Article
Urine sampling technique	Curtis P, Kim-Foley S, Kebede M	1993	References	Commentary/review	Article
Evaluation of urine sampling technique: bacterial contamination of samples from women students	Baerheim A (1), Digranes A, Hunskaar S.	1992	References	Wrong group	Article
Bacteriological findings in urine specimens from women. Association with urinary tract symptoms and sampling methods.	Baerheim A, Digranes A, Hunskaar S, Laerum E.	1991	Medline	Wrong design	Abstract
Perineal cleansing and midstream urine specimens in ambulatory women	Holliday G, Strike PW, Masterton RG	1991	References	Wrong group	Article
Urine sampling in ambulatory women.	Walter FG	1990	Medline	Duplicate	Abstract
An approach to urinary tract infections in ambulatory women	Ronald AR, Conway B	1988	References	Commentary/review	Article
Laboratory diagnosis of urinary tract infection in ambulatory women.	Latham R.H. Wong E.S. Larson A.	1985	Embase	Wrong design	Abstract
Clean-catch versus straight-catheter urinalysis results in women.	Guss DA, Dunford JV, Griffith LD, Neuman TS, Baxt WG, Winger B, Gruber SL.	1985	Medline	Wrong design	Article
Validity of urinary catheter specimen for diagnosis of urinary tract infection in the elderly.	Grahn D, Norman DC, White ML, Cantrell M, Yoshikawa TT.	1985	Medline	Wrong group	Abstract
Is the Clean-Catch Midstream Void Procedure Necessary for Obtaining Urine Culture Specimens from Men?	Lipsky BA, Inui TS, Plorde JJ, Berger RE.	1984	References	Wrong group	Article
Comparison of mid catheter collection and suprapubic aspiration of urine for diagnosing bacteriuria due to fastidious micro-organisms.	Savige JA, Birch DF, Fairley KF.	1983	Medline	Wrong setting	Article
THE MYTH OF THE CLEAN CATCH URINE SPECIMEN	Immergut MA, Gilbert EC, Frensilli FJ, Goble M	1981	References	Wrong group	Article
[Diagnosis of urinary infections by the transportable agar method. Collection of urine in non-sterile containers].	Svendsen I, Eklund A.	1980	Medline	Wrong design	Article
An automatic midstream urine collector.	King MR.	1980	Medline	Wrong setting	Article
Perineal cleansing before midstream urine, a necessary ritual.	Morris RW, Watts MR, Reeves DS.	1979	Medline	Missing data	Article
Comparison of paired midstream voided and catheterized urine samples from female patients in a general hospital.	Barnes WF, Albers DD.	1978	Medline	Wrong group	Article
New method for obtaining uncontaminated urine from women.	Cade R, Raulerson JD, Mahoney JJ, Duprey P, Privette M, Phelan MC, Beers H, Fuller TJ, Juncos LI, Grubb WG.	1978	Medline	Wrong group	Abstract
Comparison of paired midstream voided and catheterized urine sam- ples from female patients in a general hospital.	Barnes WF, Albers DD	1978	References	Wrong group	Article
Qualitative assessment of midstream urine cultures in the detection of bacteriuria.	Gower P.E. Roberts A.P.	1975	Embase	Wrong group	Abstract
Bacterial contamination of urine, collected in fractions from different phases of micturition. A study in healthy women.	Henning C, Tornvall G.	1975	Medline	Wrong group	Abstract
Correlation of a new urine collection-culture tube with the standard loop technique.	Martin LP, Ahmed M.	1974	Medline	Wrong setting	Article
[Examination of the urine. Collection, transport and quantitative bacteriological assessment].	Vejlsgaard R.	1969	Medline	Commentary/review	Article
Suprapubic bladder aspiration in diagnosis of urinary tract infection.	Bailey RR, Little PJ.	1969	Medline	Wrong design	Article
Reliability of clean-voided mid-stream urine specimens for the diagnosis of significant bacteriuria in the female patient.	Lemieux G, St-Martin M.	1968	Medline	Wrong setting	Article
Voided urine cultures in women. A study of 425.	Breitenbucher BR.	1966	Medline	Wrong setting	Article
The collection of urine for bacteriological investigation.	Craig I.	1965	Medline	Wrong setting	Article
The collection and assessment of midstream urine samples in the diagnosis of urinary tract infection in women.	DAWBORN JK, PLUNKETT PJ.	1963	Medline	Wrong setting	Article
A screening method for the evaluation of urinary tract infections in female patients without catheterization.	BOSHELL BR, SANFORD JP	1958	Medline	Wrong group	Article
Sterile-voided urine culture; an evaluation in 100 consecutive hospitalized women.	MERRITT AD, SANFORD JP.	1958	Medline	Wrong setting	Article
A comparison of bac- terial counts of the urine obtained by needle aspiration of the bladder, catheter- ization and midstream-voided methods	Monzon OT, Ory EM, Dobson HL, Carter E, Yow EM	1958	References	Wrong setting	Article
A screening method for the evaluation of urinary tract infections in female patients without catheterization	Boswell BR, Sanford JP	1958	References	Wrong setting	Article
The case against the catheter	BEESON, P B	1958	References	Commentary/review	Article
Observations on the reliability and safety of bladder catheterization for bacteriologic study of the urine.	BEESON, P B	1956	References	Wrong group	Article

## Additional file

Additional file 1: Quadas-2. (PDF 427 kb)
