# Supplementary material for: Urine sampling techniques in symptomatic primary-care patients: a diagnostic accuracy review
Source: BMC Fam Pract. 2016 Jun 8;17:72. doi: 10.1186/s12875-016-0465-4 (PMC4898352; doi:10.1186/s12875-016-0465-4)
Supplement: Additional file 1: — Quadas-2. (PDF 427 kb) [file 12875_2016_465_MOESM1_ESM.pdf]

# QUADAS-2

| ID: | 1 | Author: | Walter FG | Year: | 1989 | Reviewer: | Anne |
|---|---|---|---|---|---|---|---|

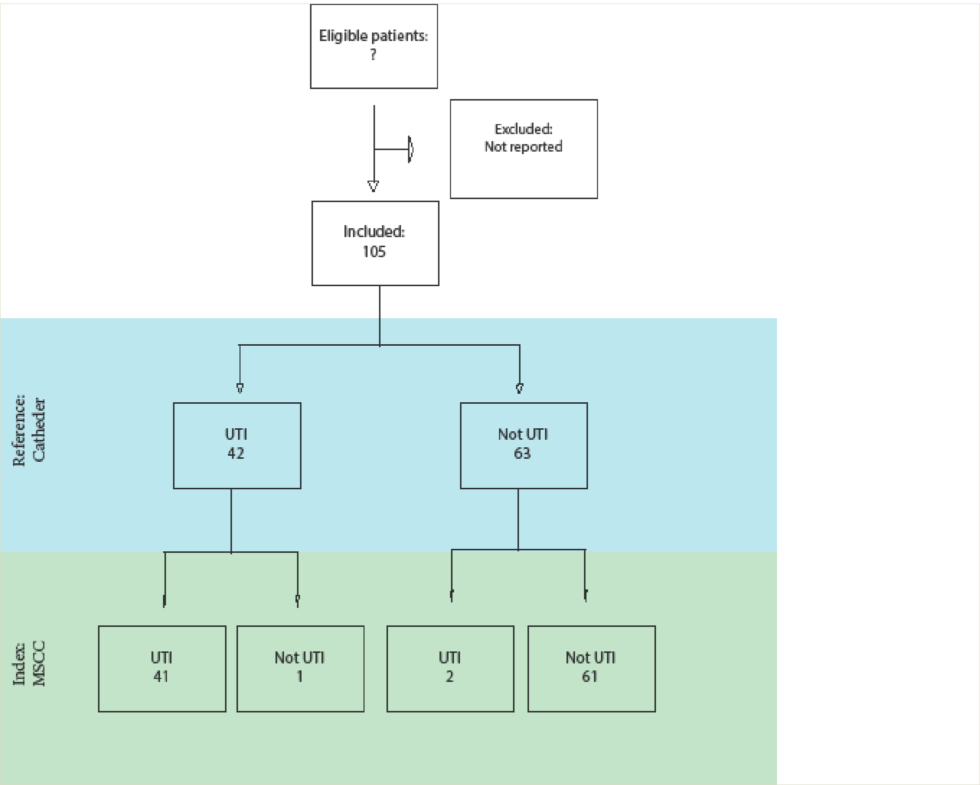


## DOMAIN 1: PATIENT SELECTION

### A. RISK OF BIAS

**Describe methods of patient selection:**

The study is defined as prospective and the patients as consequetive and symptomatic. Exclusion criteria are relevant for the study aim. The inclusion period seems appropriate for the included number of patients.

| | |
|---|---|
| Was a consecutive or random sample of patients enrolled? | Yes |
| Was a case-control design avoided? | Yes |
| Did the study avoid inappropriate exclusions?: | Yes |

**Could the selection of patients have introduced bias?** **Risk of bias:** Low

### B. APPLICABILITY:

**Describe included patients:**

Symptomatic women presenting to the triage nurse in an outpatient clinic.

**Do the included patients and setting match the question?** **Concerns regarding applicability:** Low

# QUADAS-2

| ID: | 1 | Author: | Walter FG | Year: | 1989 | Reviewer: | Anne |
|---|---|---|---|---|---|---|---|

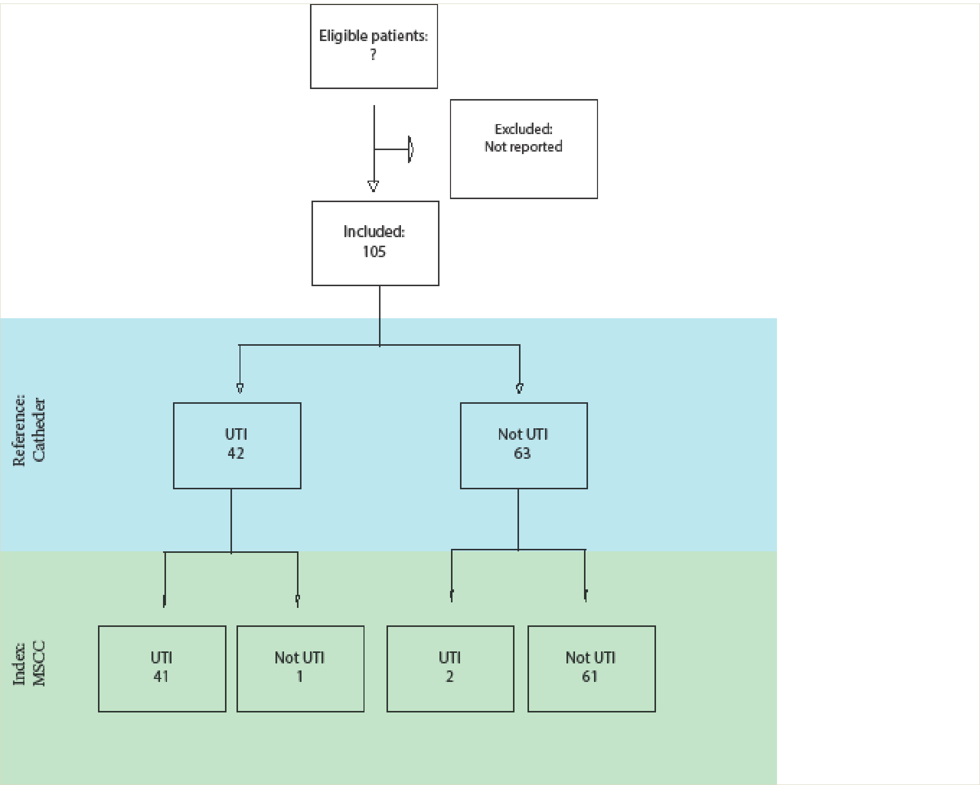


## DOMAIN 2: INDEX TEST

### A. RISK OF BIAS

**Describe the index test and how it was conducted and interpreted**

MSCC urine with prior cleansing with povidone-iodine-soaked 4 x 4 gauzes.
A positive urine culture was defined as more than 10,000 cfu/ml of urine. This definition did not include mixed flora.
A negative urine culture was defined as a sterile culture or a culture that grew mixed flora or less than 10000 cfu/ml. It is not described if the interpretor was blinded to the result of the reference. The cut-off of 10000 cfu/ml is considered clinically relevant since it has been the clinical cut-off for a long time and still is for some species.

| | |
|---|---|
| Were the index test results interpreted without knowledge of the results of the reference standard? | Unclear |
| If a threshold was used, was it pre-specified? | Yes |
| **Could the conduct or interpretation of the index test have introduced bias?** **Risk of bias:** | Unclear |

### B. APPLICABILITY:

| | |
|---|---|
| **Is there concern that the index test, its conduct, or interpretation differ fromt he review question** **Concerns regarding applicability:** | Low |

# QUADAS-2

| ID: | 1 | Author: | Walter FG | Year: | 1989 | Reviewer: | Anne |
|---|---|---|---|---|---|---|---|

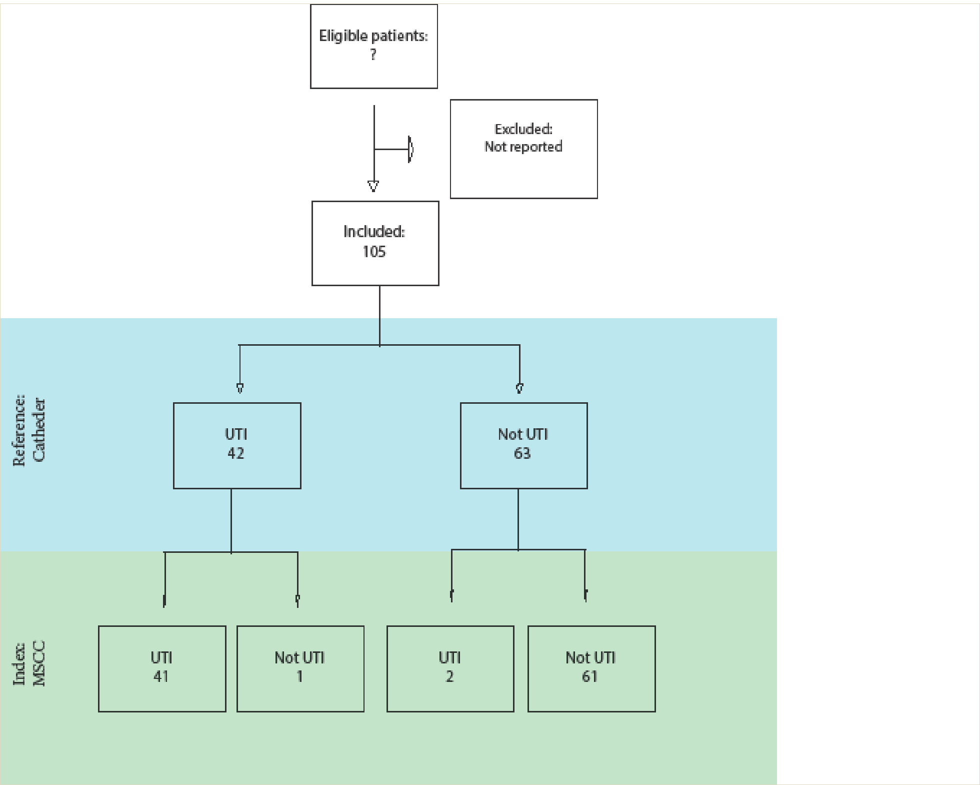


## DOMAIN 3: REFERENCE STANDARD

### A. RISK OF BIAS

**Describe the reference standard and how it was conducted and interpreted::**

Catheter urine using a Davol Single Use Female Catheterization Kit ®. A positive urine culture was defined as more than 10,000 cfu/ml of urine. This definition did not include mixed flora. A negative urine culture was defined as a sterile culture or a culture that grew mixed flora or less than 10000 cfu/ml. It is not described if the interpretor was blinded to the result of the index test. The cut-off of 10000 cfu/ml is considered clinically relevant since it has been the clinical cut-off for a long time and still is for some species.

| | |
|---|---|
| Is the reference standard likely to correctly classify the target condition? | Yes |
| Were the reference standard results interpreted without knowledge of the results of the index test? | Unclear |

**Could the reference standard, its conduct, or its interpretation have introduced bias?** **Risk of bias:** Unclear

### B. APPLICABILITY:

**Is there concern that the target condition as defined by the reference standard does not match the review question?** **Concerns regarding applicability:** Low

# QUADAS-2

| ID: | 1 | Author: | Walter FG | Year: | 1989 | Reviewer: | Anne |
|---|---|---|---|---|---|---|---|

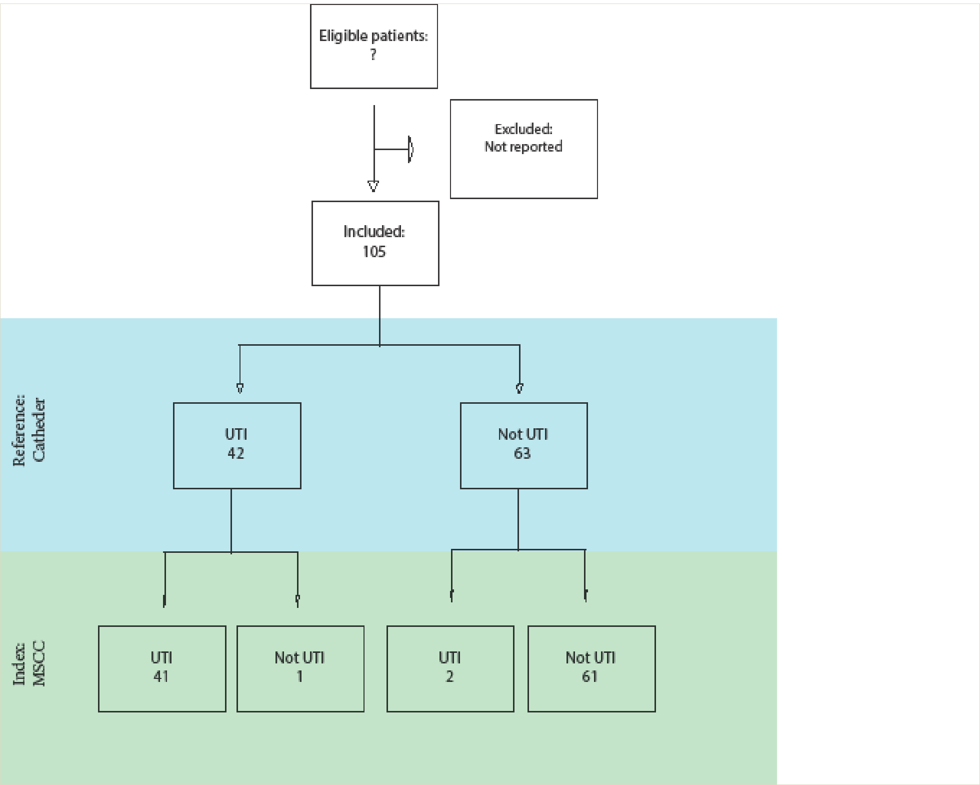


## DOMAIN 4: FLOW AND TIMING

### A. RISK OF BIAS

**Describe any patients who did not receive the index test(s) and or reference standard or who were excluded from the 2x2 table (refer to flow diagram):**

Not reported. Risk of bias is unclear since it is not reported who were excluded, but it seems unlikely a completely consecutive sample has been enrolled. All patients included were analyzed.

**Describe the time interval and any interventions between index test(s) and reference standard:**

Samles were handled simoultanously and delay was avoided.

| | |
|---|---|
| Was there an appropriate interval between index test and reference standard? | Yes |
| Did all patients receive a reference standard? | Yes |
| Did patients receive the same reference standard? | Yes |
| Were all patients included in the analysis? | Yes |

**Could the patient flow have introduced bias?** Unclear

# QUADAS-2

| ID: | 2 | Author: | Mabeck | Year: | 1969 | Reviewer: | Anne |
|---|---|---|---|---|---|---|---|

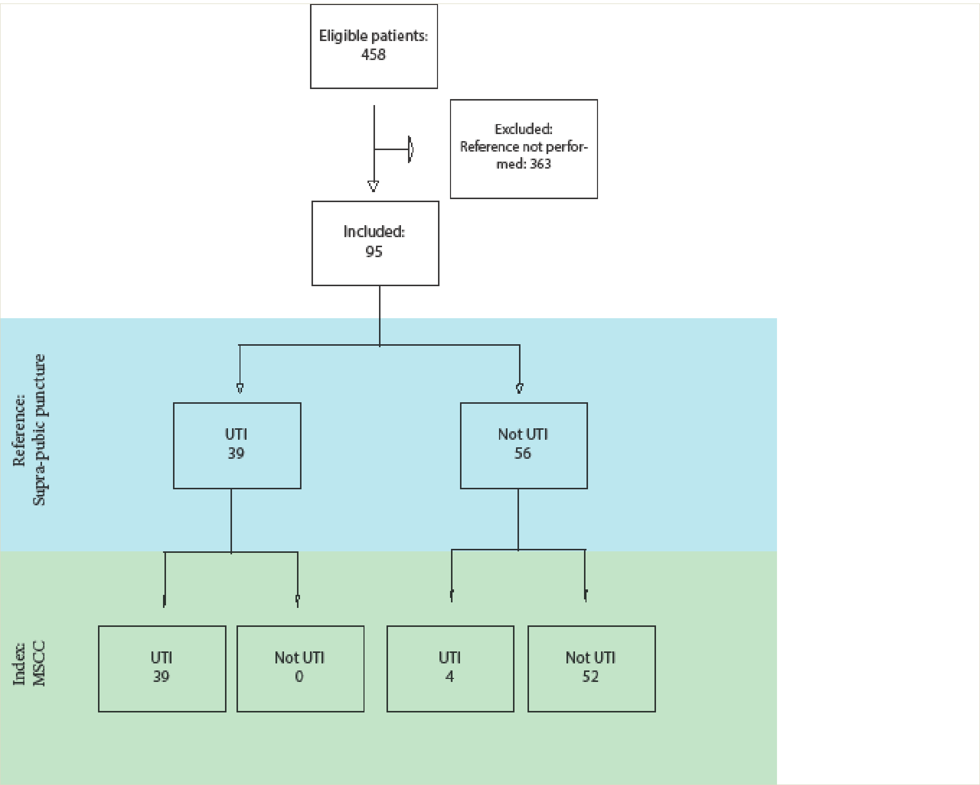


## DOMAIN 1: PATIENT SELECTION

### A. RISK OF BIAS

**Describe methods of patient selection:**

Adult females aged 16-65 referred to an outpatient clinic during 18 months with symptoms of UTI. Suprapubic puncture was only attempted last 12 months of the study, if the woman had not urinated during past 2 hours and it is not described if it failed in some cases. It is not described if the included patients were consecutive and exclusion criteria are not described.

| | |
|---|---|
| Was a consecutive or random sample of patients enrolled? | Unclear |
| Was a case-control design avoided? | Yes |
| Did the study avoid inappropriate exclusions?: | Unclear |

**Could the selection of patients have introduced bias?** **Risk of bias:** Unclear

### B. APPLICABILITY:

**Describe included patients:**

Patients are female and have symptoms, otherwice not described.

**Do the included patients and setting match the question?** **Concerns regarding applicability:** Low

# QUADAS-2

| ID: | 2 | Author: | Mabeck | Year: | 1969 | Reviewer: | Anne |
|---|---|---|---|---|---|---|---|

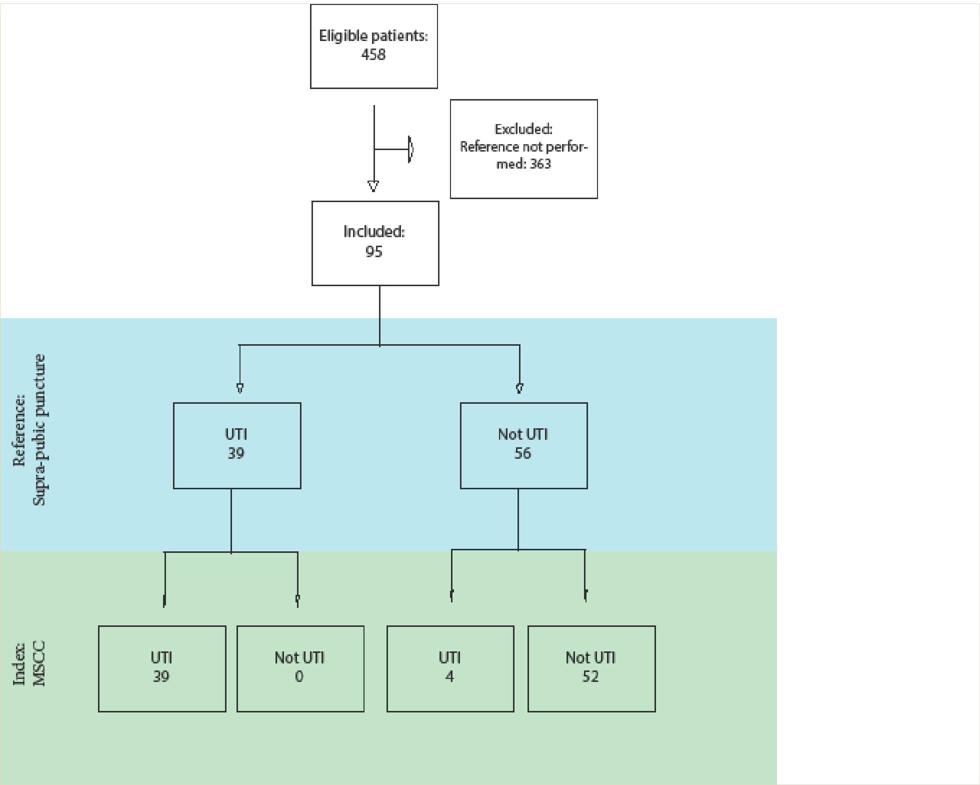


## DOMAIN 2: INDEX TEST

**A. RISK OF BIAS**

**Describe the index test and how it was conducted and interpreted**

The patients were instructed and washed by a nurse and vagina plugged with cotton-swab. Afterwards the patient voided and a plastic cup was introduced mid-stream and the sample refrigerated immediately.
No threshold reported. It is unclear if the aim from the beginning was to report the absolute numbers, but probably. Since data can be obtained about any cut-off, concerns about applicability are low

| | |
|---|---|
| Were the index test results interpreted without knowledge of the results of the reference standard? | Unclear |
| If a threshold was used, was it pre-specified? | Unclear |

**Could the conduct or interpretation of the index test have introduced bias?** **Risk of bias:** Unclear

**B. APPLICABILITY:**

**Is there concern that the index test, its conduct, or interpretation differ fromt he review question** **Concerns regarding applicability:** Low

# QUADAS-2

| ID: | 2 | Author: | Mabeck | Year: | 1969 | Reviewer: | Anne |
|---|---|---|---|---|---|---|---|

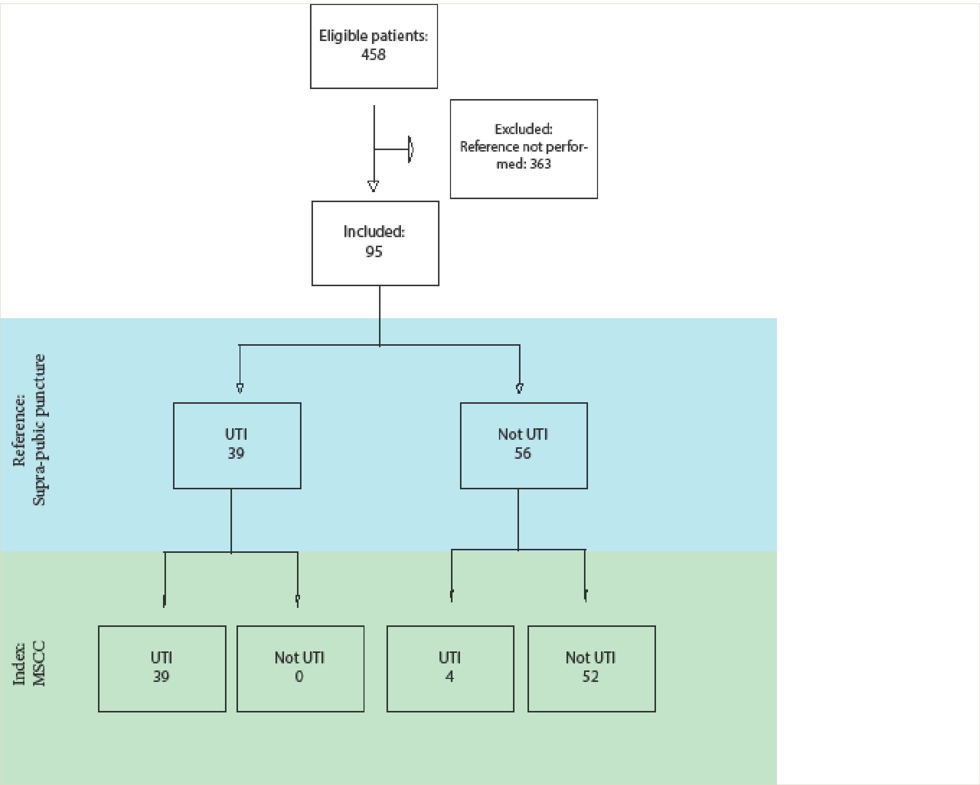


## DOMAIN 3: REFERENCE STANDARD

### A. RISK OF BIAS

**Describe the reference standard and how it was conducted and interpreted::**

Suprapubic puncture was attemped on the second visit 1 day after index test. No threshold reported.

| | |
|---|---|
| Is the reference standard likely to correctly classify the target condition? | Yes |
| Were the reference standard results interpreted without knowledge of the results of the index test? | Unclear |

**Could the reference standard, its conduct, or its interpretation have introduced bias?** **Risk of bias:** Unclear

### B. APPLICABILITY:

**Is there concern that the target condition as defined by the reference standard does not match the review question?** **Concerns regarding applicability:** Low

# QUADAS-2

| ID: | 2 | Author: | Mabeck | Year: | 1969 | Reviewer: | Anne |
|---|---|---|---|---|---|---|---|

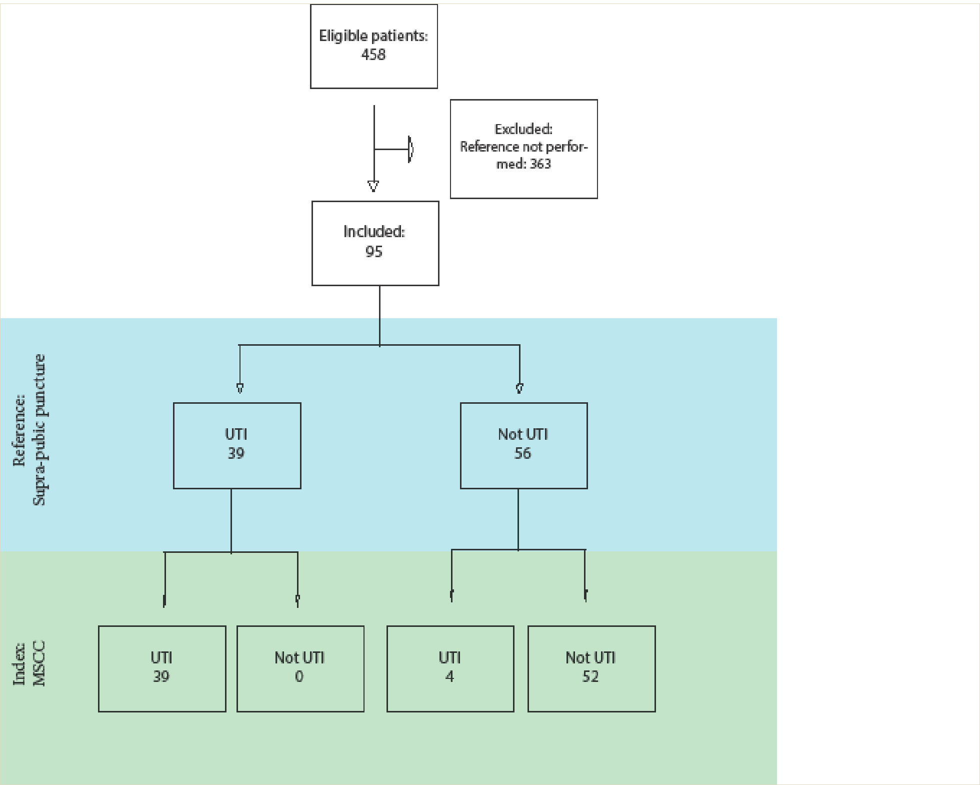


## DOMAIN 4: FLOW AND TIMING

### A. RISK OF BIAS

**Describe any patients who did not receive the index test(s) and or reference standard or who were excluded from the 2x2 table (refer to flow diagram):**

363 /458 were excluded because no suprapibic puncture was made. It was not described if most of these were during the first 6 months when it was not attempted or most were due to an empty bladder or other causes. Risk of bias high due to this and to the interval between index and reference

**Describe the time interval and any interventions between index test(s) and reference standard:**

1 day

| | |
|---|---|
| Was there an appropriate interval between index test and reference standard? | No |
| Did all patients receive a reference standard? | Yes |
| Did patients receive the same reference standard? | Yes |
| Were all patients included in the analysis? | Yes |
| **Could the patient flow have introduced bias?** | High |

# QUADAS-2

| ID: | 3 | Author: | Hooton | Year: | 2013 | Reviewer: | Anne |
|---|---|---|---|---|---|---|---|

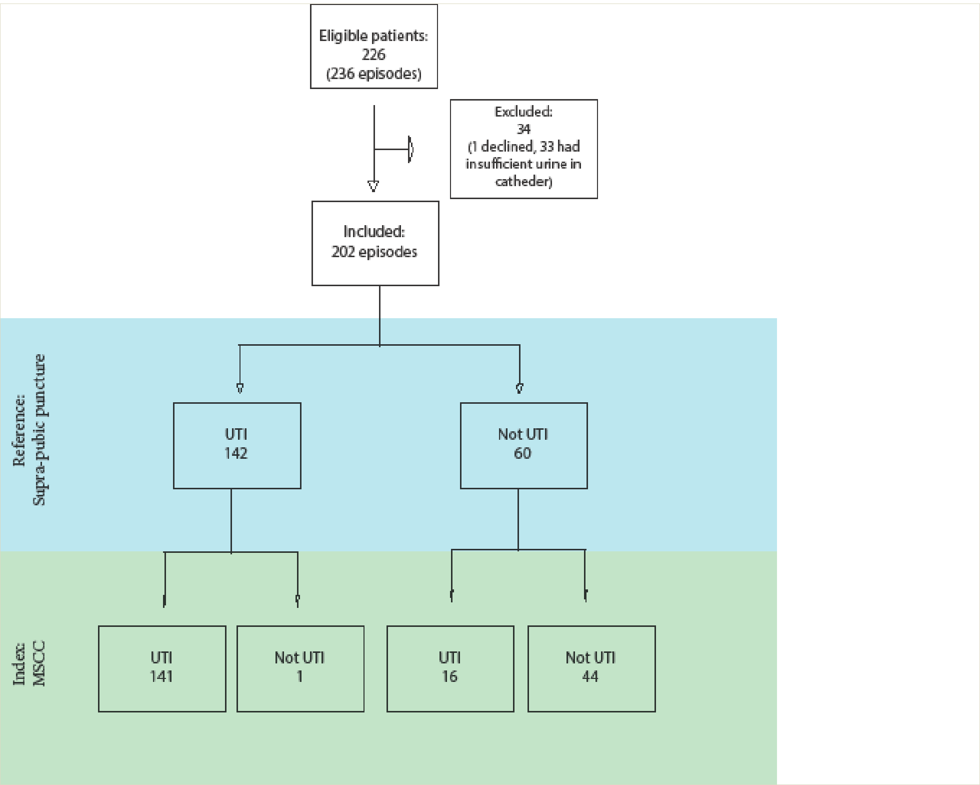


## DOMAIN 1: PATIENT SELECTION

### A. RISK OF BIAS

**Describe methods of patient selection:**

Patients recruited though ads and flyers. Less than 202 patients included despite an inclusion period of 10 years. This can not be a consequetive or random sample. Risk of bias therefore high. Exclusion criteria relevant.

| | |
|---|---|
| Was a consecutive or random sample of patients enrolled? | No |
| Was a case-control design avoided? | Yes |
| Did the study avoid inappropriate exclusions?: | Yes |

**Could the selection of patients have introduced bias?** **Risk of bias:** Low

### B. APPLICABILITY:

**Describe included patients:**

Patients were female and symptomatic and were recruited from primary care. The sample is, however, very selected since the study was not conducted in primary care and we assume the patients are not completly comparable to primary care patients.

**Do the included patients and setting match the question?** **Concerns regarding applicability:** High

# QUADAS-2

| ID: | 3 | Author: | Hooton | Year: | 2013 | Reviewer: | Anne |
|---|---|---|---|---|---|---|---|

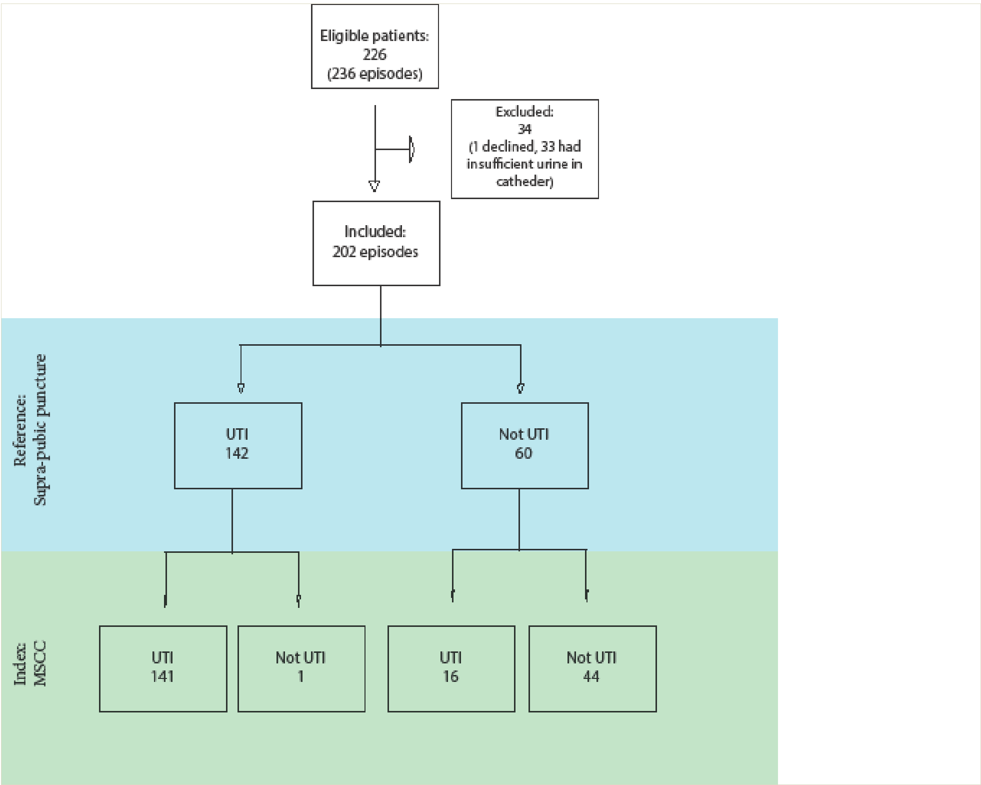


## DOMAIN 2: INDEX TEST

### A. RISK OF BIAS

**Describe the index test and how it was conducted and interpreted**

MSCC sample with prior cleaning with castile soap towelettes. Cut-off for uropathogens 10 cfu/ml. It is unclear if the intepretor was blinded to the result of the index test.

| | |
|---|---|
| Were the index test results interpreted without knowledge of the results of the reference standard? | Unclear |
| If a threshold was used, was it pre-specified? | Yes |

**Could the conduct or interpretation of the index test have introduced bias?** **Risk of bias:** Unclear

### B. APPLICABILITY:

**Is there concern that the index test, its conduct, or interpretation differ fromt he review question** **Concerns regarding applicability:** Low

# QUADAS-2

| ID: | 3 | Author: | Hooton | Year: | 2013 | Reviewer: | Anne |
|---|---|---|---|---|---|---|---|

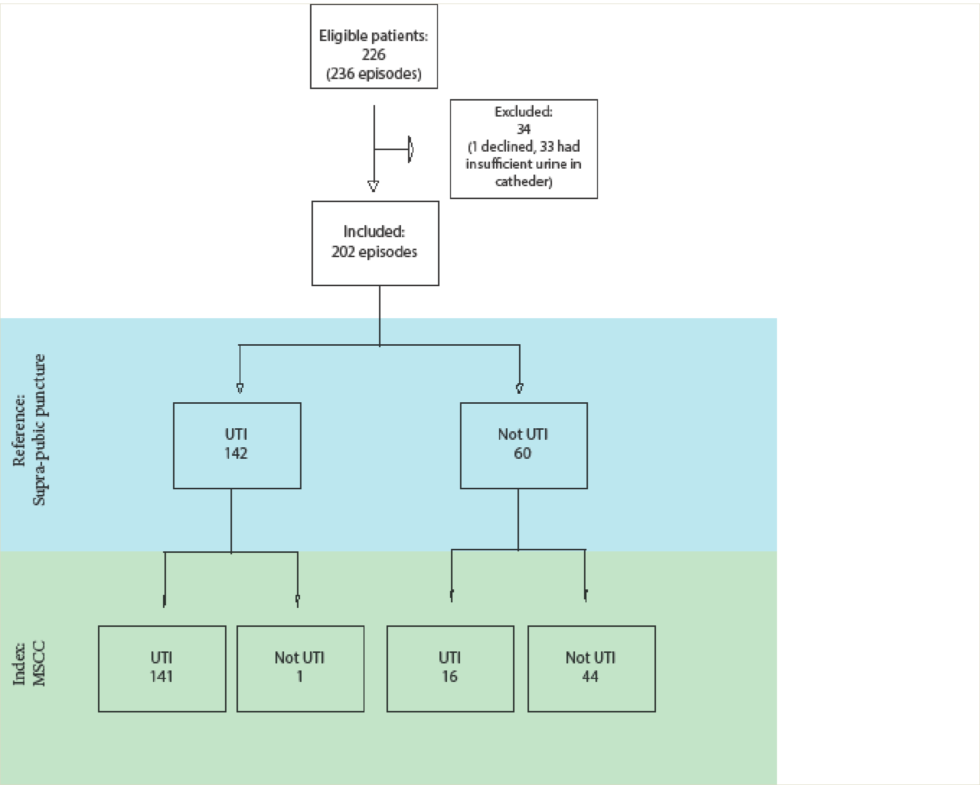


## DOMAIN 3: REFERENCE STANDARD

### A. RISK OF BIAS

**Describe the reference standard and how it was conducted and interpreted::**

After MSCC, a catheder sample with a french urethral catheter. Cut-off for uropathogens 10 cfu/ml. . It is unclear if the intepretor was blinded to the result of the index test.

| | |
|---|---|
| Is the reference standard likely to correctly classify the target condition? | Yes |
| Were the reference standard results interpreted without knowledge of the results of the index test? | Unclear |

**Could the reference standard, its conduct, or its interpretation have introduced bias?** **Risk of bias:** Unclear

### B. APPLICABILITY:

**Is there concern that the target condition as defined by the reference standard does not match the review question?** **Concerns regarding applicability:** Low

# QUADAS-2

| ID: | 3 | Author: | Hooton | Year: | 2013 | Reviewer: | Anne |
|---|---|---|---|---|---|---|---|

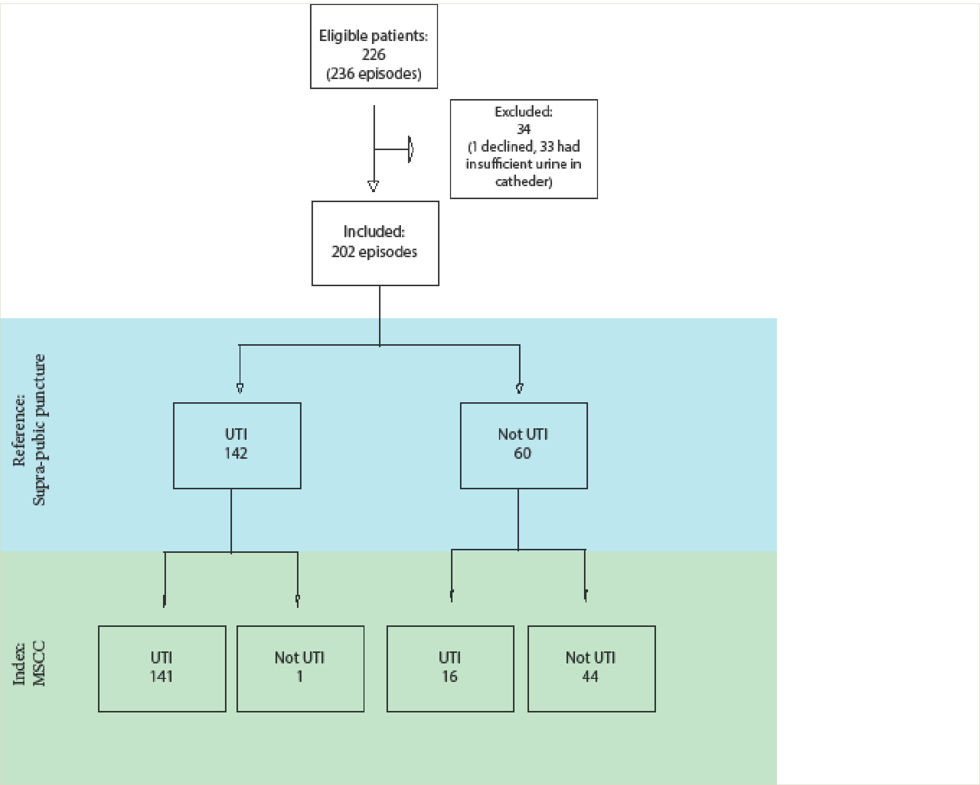


## DOMAIN 4: FLOW AND TIMING

### A. RISK OF BIAS

**Describe any patients who did not receive the index test(s) and or reference standard or who were excluded from the 2x2 table (refer to flow diagram):**

Of the included patients, all took part in the analysis. 34 were excluded because the reference
The study includes samples in their ananlysis not patients. The number of dublicates is asumed low allthough they dont report the number for the final 202 patients. Risk of bias is set to low.

**Describe the time interval and any interventions between index test(s) and reference standard:**

Same time, fine

| | |
|---|---|
| Was there an appropriate interval between index test and reference standard? | Yes |
| Did all patients receive a reference standard? | Yes |
| Did patients receive the same reference standard? | Yes |
| Were all patients included in the analysis? | Yes |

**Could the patient flow have introduced bias?** Low

# QUADAS-2

ID: **4** | Author: Stamm | Year: 1982 | Reviewer: **Anne**

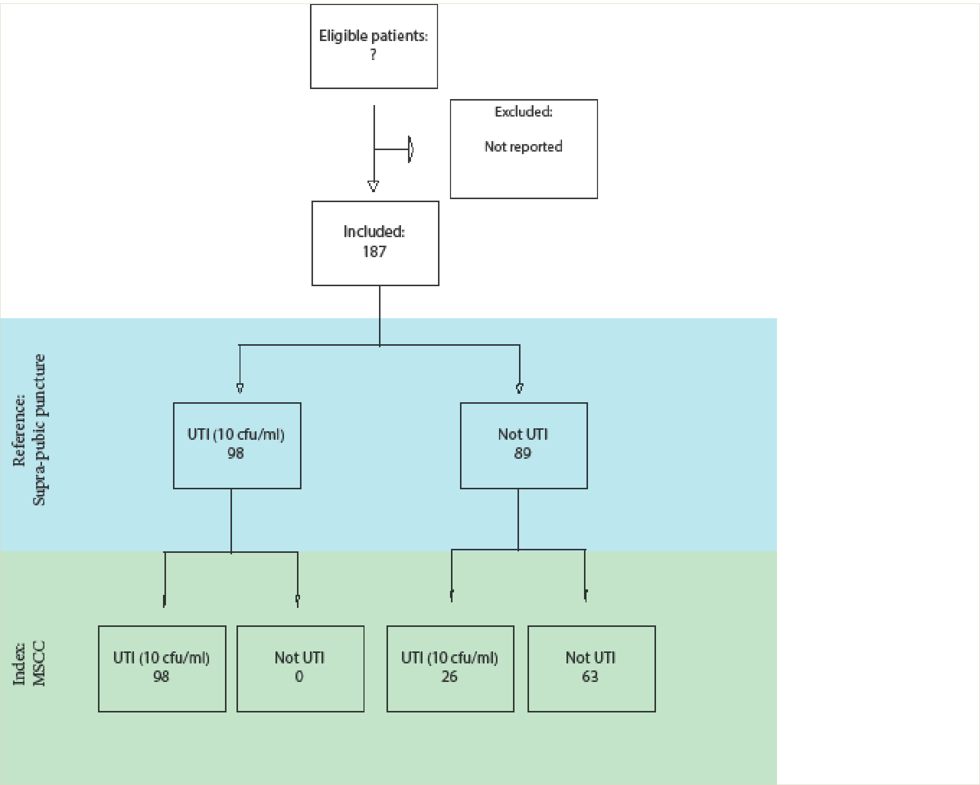


## DOMAIN 1: PATIENT SELECTION

### A. RISK OF BIAS

**Describe methods of patient selection:**

Women referred to outpatient clinic based on dysuria and frequency. Exclusion criteria relevant except that genital examination was performed first and patients excluded if there were positive findings. This may affect applicability. It is not reported if the patients were consecutive

| | |
|---|---|
| Was a consecutive or random sample of patients enrolled? | Unclear |
| Was a case-control design avoided? | Yes |
| Did the study avoid inappropriate exclusions?: | Yes |

**Could the selection of patients have introduced bias?** **Risk of bias:** Unclear

### B. APPLICABILITY:

**Describe included patients:**

Women referred til outpatient clinic based on dysuria and frequency. Also excluded if genital infection after genital examination. This is not usual clinical practice and it could affect the applicability.

**Do the included patients and setting match the question?** **Concerns regarding applicability:** Unclear

# QUADAS-2

| ID: | 4 | Author: | Stamm | Year: | 1982 | Reviewer: | Anne |
|---|---|---|---|---|---|---|---|

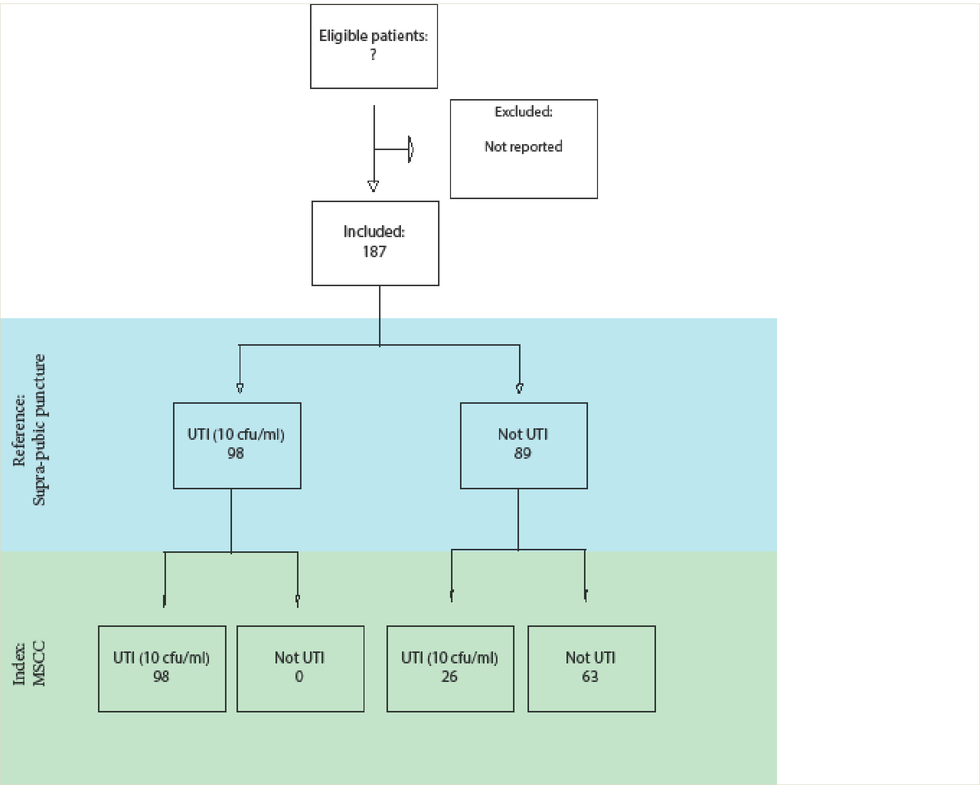


## DOMAIN 2: INDEX TEST

**A. RISK OF BIAS**

**Describe the index test and how it was conducted and interpreted**

Morning urine or more than 4 hours incubation time. Catheter or suprapubic aspiration first, then first-void urine and then MSCC urine. Samples refrigerated and transported to the lab within 4 hours. Coliform colonies identified and quantified in absolute numbers. It is unclear if the interpreter was blinded to the result of the index test. Due to the study design where a GE, several genital swabs and a catheterization were performed before the urine sample, our concerns regarding applicability are high since it is likely the procedures may have introduced bacteria.

| | |
|---|---|
| Were the index test results interpreted without knowledge of the results of the reference standard? | Unclear |
| If a threshold was used, was it pre-specified? | Yes |

**Could the conduct or interpretation of the index test have introduced bias?** **Risk of bias:** Unclear

**B. APPLICABILITY:**

**Is there concern that the index test, its conduct, or interpretation differ fromt he review question** **Concerns regarding applicability:** High

# QUADAS-2

| ID: | 4 | Author: | Stamm | Year: | 1982 | Reviewer: | Anne |
|---|---|---|---|---|---|---|---|

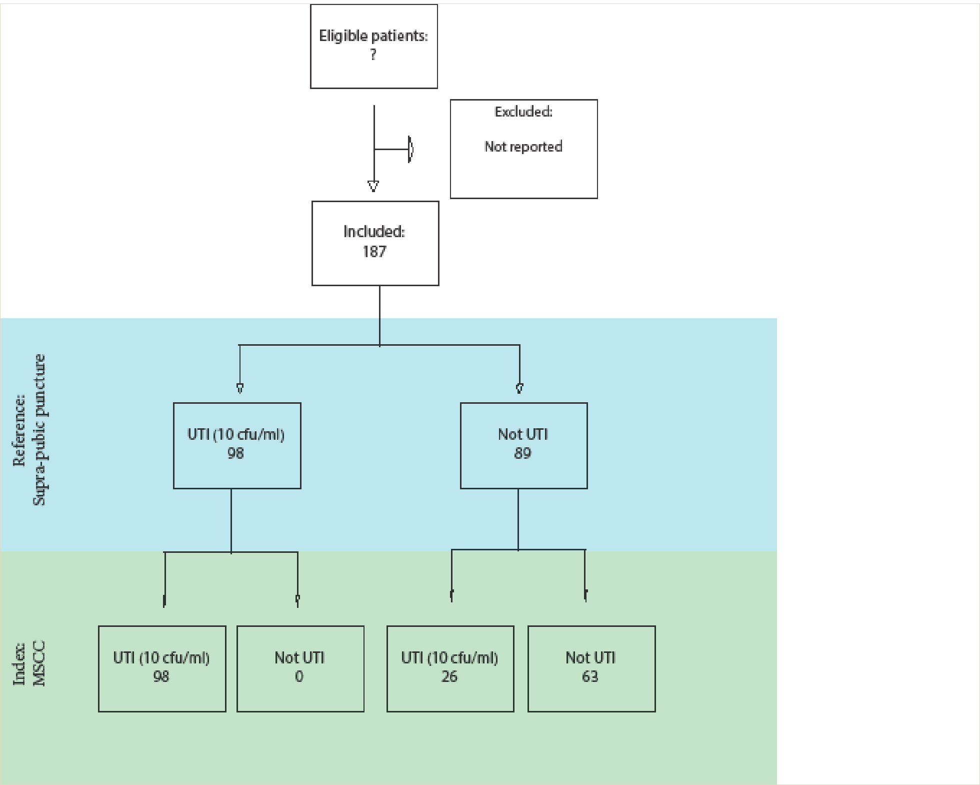


## DOMAIN 3: REFERENCE STANDARD

**A. RISK OF BIAS**

**Describe the reference standard and how it was conducted and interpreted::**

Morning urine or more than 4 hours incubation time. Catheter or suprapubic aspiration first, then first-void urine and then MSCC urine. Samples refrigerated and transported to the lab within 4 hours. Coliform colonies identified and quantified in absolute numbers. 10 cfu/ml cutoff for all samples both catheter and suprapubic puncture. In 66 women the index was only taken with a second sample 18-72 hours after the women consulted. Some references were with suprapubic puncture and some with catheter. It is unclear if the interpreter was blinded to the result of the index test.

| | |
|---|---|
| Is the reference standard likely to correctly classify the target condition? | Yes |
| Were the reference standard results interpreted without knowledge of the results of the index test? | Unclear |

**Could the reference standard, its conduct, or its interpretation have introduced bias?** **Risk of bias:** Unclear

**B. APPLICABILITY:**

**Is there concern that the target condition as defined by the reference standard does not match the review question?** **Concerns regarding applicability:** Low

# QUADAS-2

| ID: | 4 | Author: | Stamm | Year: | 1982 | Reviewer: | Anne |
|---|---|---|---|---|---|---|---|

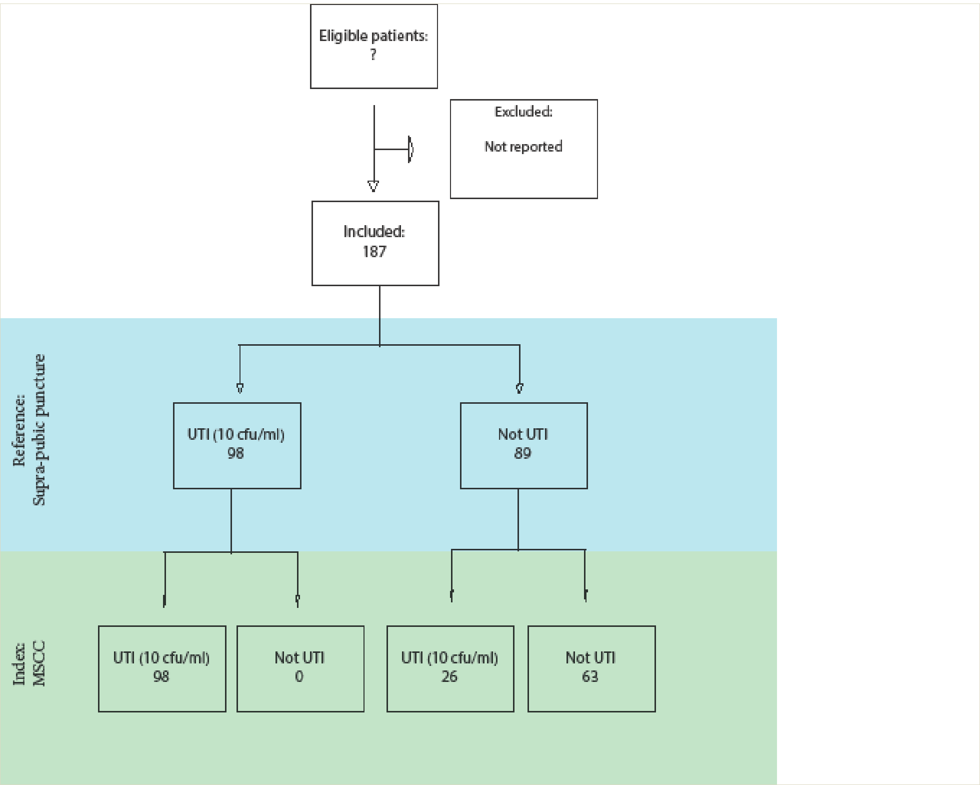


## DOMAIN 4: FLOW AND TIMING

### A. RISK OF BIAS

**Describe any patients who did not receive the index test(s) and or reference standard or who were excluded from the 2x2 table (refer to flow diagram):**

It is not reported who were excluded, but it seems unlikely a completely consecutive sample has been enrolled. All patients included were analyzed. Because of this and the two different references used, risk of bias is set to high even though the two references have no consequences in our study

**Describe the time interval and any interventions between index test(s) and reference standard:**

They do not report if there is any delay between the samples. It is assumed it is from the same urine, but not clear.

| | |
|---|---|
| Was there an appropriate interval between index test and reference standard? | Yes |
| Did all patients receive a reference standard? | Yes |
| Did patients receive the same reference standard? | No |
| Were all patients included in the analysis? | Yes |

**Could the patient flow have introduced bias?** High

# QUADAS-2

| ID: | 5 | Author: | Baerheim | Year: | 1990 | Reviewer: | Anne |
|---|---|---|---|---|---|---|---|

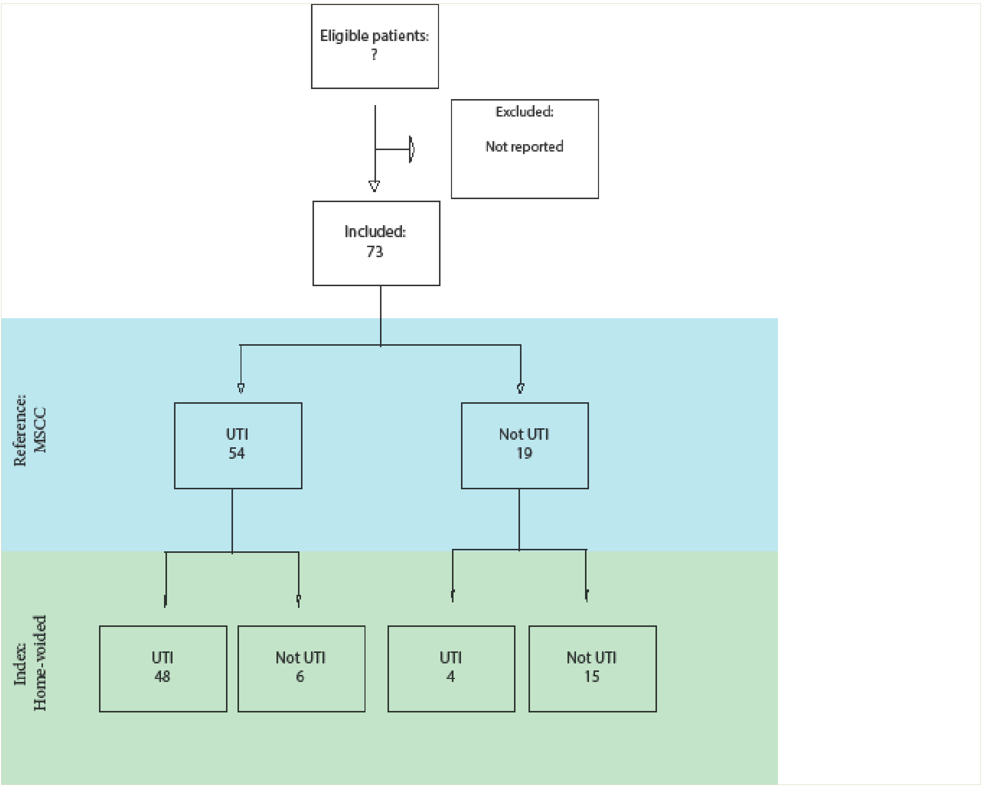


## DOMAIN 1: PATIENT SELECTION

**A. RISK OF BIAS**

**Describe methods of patient selection:**

Nineteen GPs in seven practices in western Norway recruited patients during ten months from June 1987. Exclusion criteria not described. It is unclear if the patients were consecutive.

| | |
|---|---|
| Was a consecutive or random sample of patients enrolled? | Unclear |
| Was a case-control design avoided? | Yes |
| Did the study avoid inappropriate exclusions?: | Unclear |

**Could the selection of patients have introduced bias?** **Risk of bias:** Unclear

**B. APPLICABILITY:**

**Describe included patients:**

Symptomatic female patients in general practice. This study only investigated pateints who came with a home-voided sample. They are probably not representative of the average patient in primary care. Concerns regarding applicability therefore high

**Do the included patients and setting match the question?** **Concerns regarding applicability:** High

# QUADAS-2

ID: **5** Author: Baerheim Year: 1990 Reviewer: **Anne**

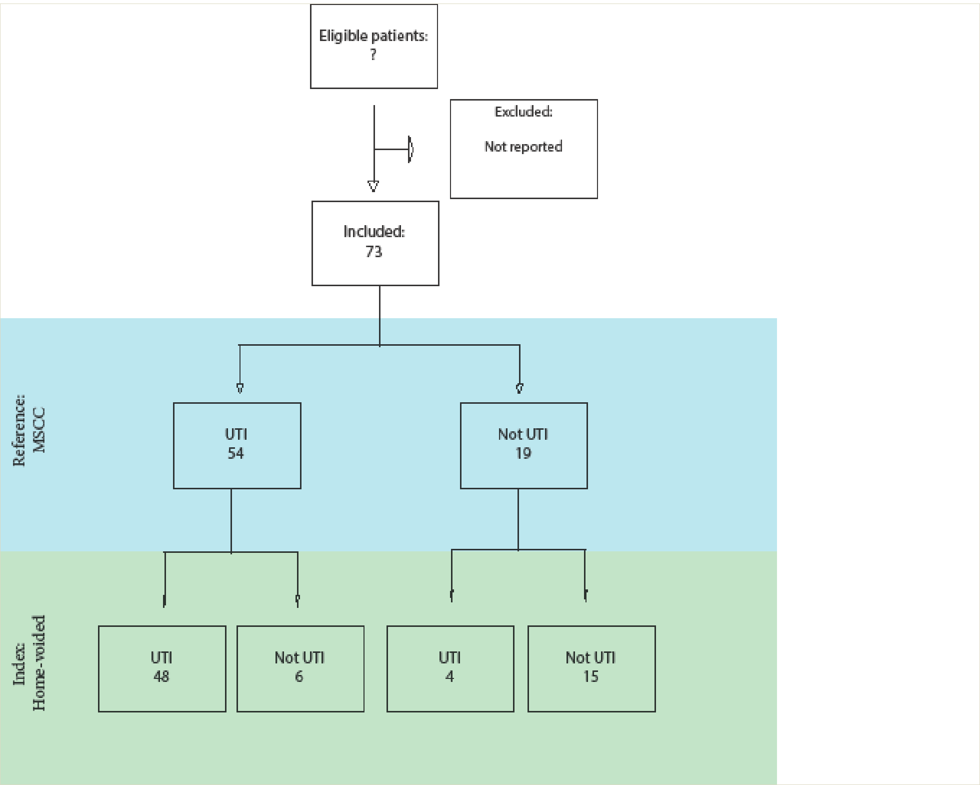


## DOMAIN 2: INDEX TEST

**A. RISK OF BIAS**

**Describe the index test and how it was conducted and interpreted**

Home-voided sample without instruction. 96% had used a cleansed container. 11,5% used the MSCC technique while taking it. Bladder incubation time was 5,4 hours and transport lag 3.1 hours. The samples were incubated on uricult media and sent to microbiology (standard procedure at the time).Bacteriuria was defined at two cut-off points, 104 and 105 cfu/ml of uropathogen bacteria, or any amount of Staphylococcus saprophyticus. This cut-off is considered close to current cut-offs and have been used before.

Were the index test results interpreted without knowledge of the results of the reference standard? Yes

If a threshold was used, was it pre-specified? Yes

**Could the conduct or interpretation of the index test have introduced bias?** **Risk of bias:** Low

**B. APPLICABILITY:**

**Is there concern that the index test, its conduct, or interpretation differ fromt he review question** **Concerns regarding applicability:** Low

# QUADAS-2

| ID: | 5 | Author: | Baerheim | Year: | 1990 | Reviewer: | Anne |
|---|---|---|---|---|---|---|---|

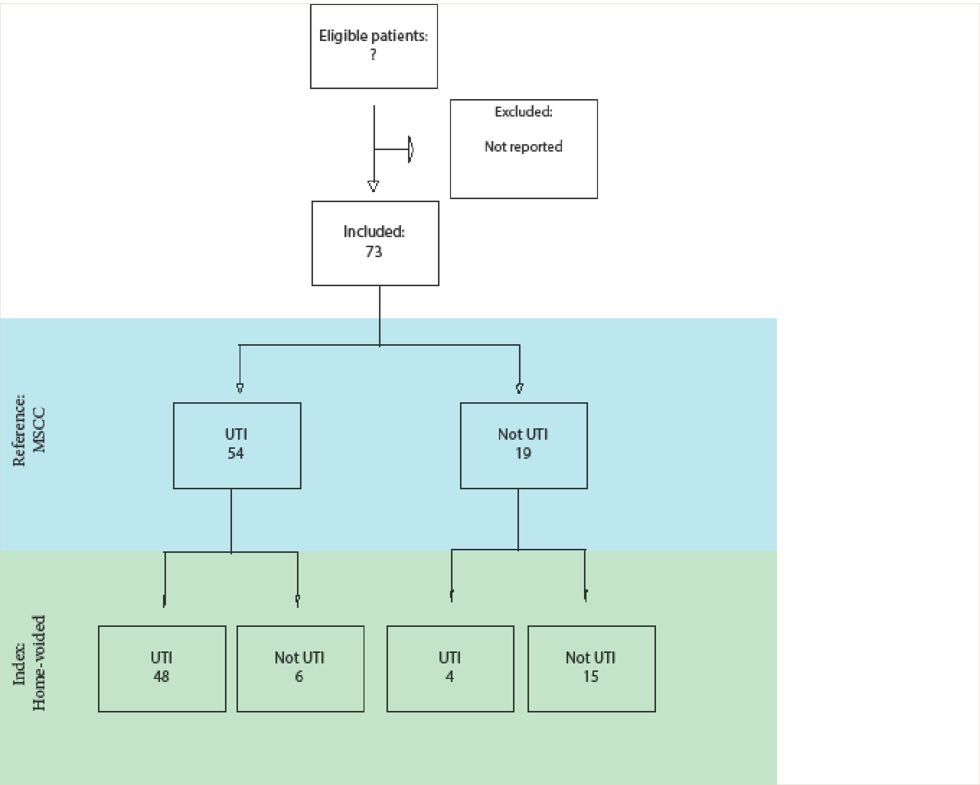


## DOMAIN 3: REFERENCE STANDARD

### A. RISK OF BIAS

**Describe the reference standard and how it was conducted and interpreted::**

MSCC with prior cleaning using sterile cotton swabs moistened with tap water. Bladder incubation time was 2.4 hours and transport lag 0 hours. The samples were incubated on uricult media and sent to microbiology (standard procedure at the time). Bacteriuria was defined at two cut-off points, 104 and 105 cfu/ml of uropathogen bacteria, or any amount of Staphylococcus saprophyticus. This cut-off is considered close to current cut-offs and have been used before.

| | |
|---|---|
| Is the reference standard likely to correctly classify the target condition? | Yes |
| Were the reference standard results interpreted without knowledge of the results of the index test? | Yes |

**Could the reference standard, its conduct, or its interpretation have introduced bias?** **Risk of bias:** Low

### B. APPLICABILITY:

**Is there concern that the target condition as defined by the reference standard does not match the review question?** **Concerns regarding applicability:** Low

# QUADAS-2

| ID: | 5 | Author: | Baerheim | Year: | 1990 | Reviewer: | Anne |
|---|---|---|---|---|---|---|---|

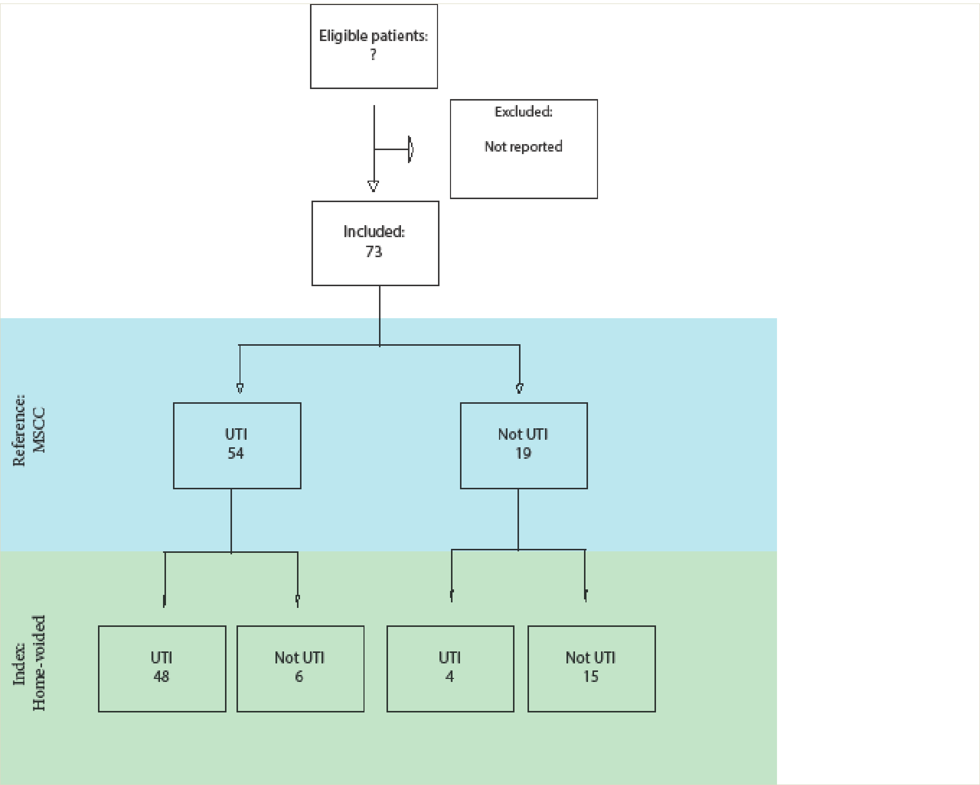


## DOMAIN 4: FLOW AND TIMING

### A. RISK OF BIAS

**Describe any patients who did not receive the index test(s) and or reference standard or who were excluded from the 2x2 table (refer to flow diagram):**

Not reported. It seems they have included a completely consecutive sample of patients bring a home-voided urine. Risk of bias is therefor set to low in this domain

**Describe the time interval and any interventions between index test(s) and reference standard:**

5-6 hours, ok

| | |
|---|---|
| Was there an appropriate interval between index test and reference standard? | Yes |
| Did all patients receive a reference standard? | Yes |
| Did patients receive the same reference standard? | Yes |
| Were all patients included in the analysis? | Yes |
| **Could the patient flow have introduced bias?** | Low |

# QUADAS-2

| ID: | 6 | Author: | Lifshitz | Year: | 2000 | Reviewer: | Anne |
|---|---|---|---|---|---|---|---|

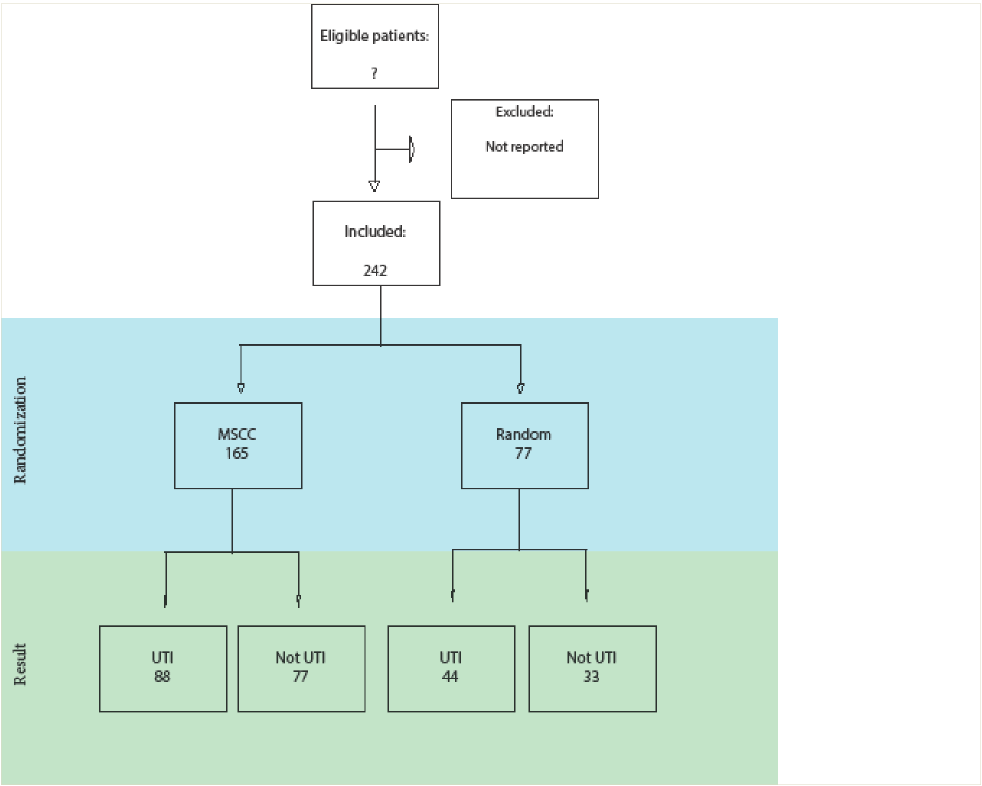


## DOMAIN 1: PATIENT SELECTION

### A. RISK OF BIAS

**Describe methods of patient selection:**

Consecutive female patients who presented to a university clinic and had symptoms suggestive of cystitis. Exclusion criteria relevant.

| | |
|---|---|
| Was a consecutive or random sample of patients enrolled? | Yes |
| Was a case-control design avoided? | Yes |
| Did the study avoid inappropriate exclusions?: | Yes |

**Could the selection of patients have introduced bias?** **Risk of bias:** Low

### B. APPLICABILITY:

**Describe included patients:**

Female patients with symptoms. Uptake in University clinic described and seems relevant for primary care.

**Do the included patients and setting match the question?** **Concerns regarding applicability:** Low

# QUADAS-2

ID: **6** Author: Lifshitz Year: 2000 Reviewer: **Anne**

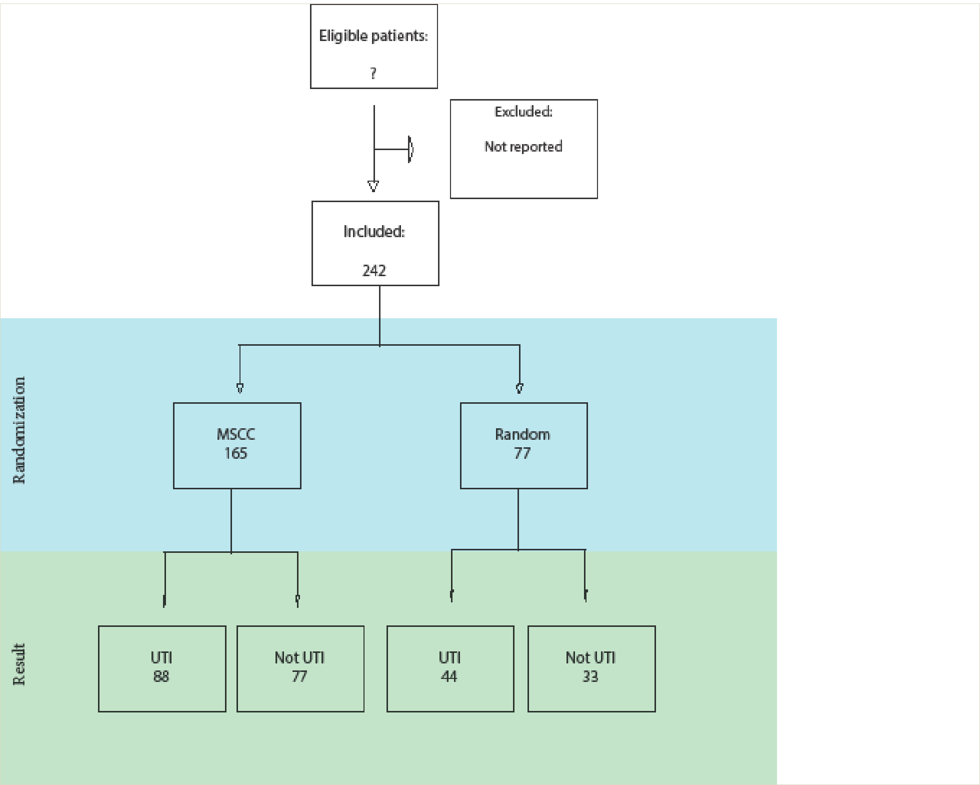


## DOMAIN 2: INDEX TEST

**A. RISK OF BIAS**

**Describe the index test and how it was conducted and interpreted**

No instructions. Urine sample sent in boric acid to the laboratory. Microbiologists at the laboratory were blinded as to grouping. Final culture reports were classified as no growth, mixed, or pure. Mixed was defined as at least 2 organisms, and in most cases, specific identification of those organisms was not made. Those that were pure were further categorized according to species and colony-forming units per milliliter using a standard technique. Coliform organisms of 102 colonyforming units per milliliter or more were considered significant.

Were the index test results interpreted without knowledge of the results of the reference standard? Yes

If a threshold was used, was it pre-specified? Yes

**Could the conduct or interpretation of the index test have introduced bias?** **Risk of bias:** Low

**B. APPLICABILITY:**

**Is there concern that the index test, its conduct, or interpretation differ fromt he review question** **Concerns regarding applicability:** Low

# QUADAS-2

| ID: | 6 | Author: | Lifshitz | Year: | 2000 | Reviewer: | Anne |
|---|---|---|---|---|---|---|---|

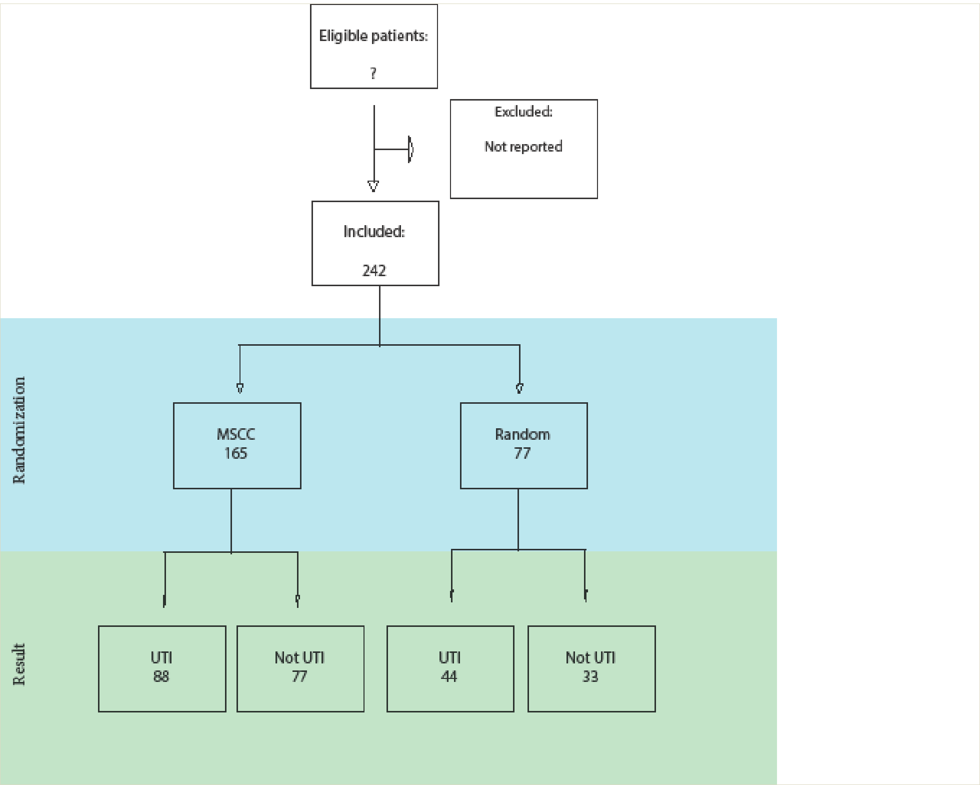


## DOMAIN 3: REFERENCE STANDARD

**A. RISK OF BIAS**

**Describe the reference standard and how it was conducted and interpreted::**

MSCC with or without a vaginal tampon. The authors have analyzed the two groups separately. The difference in references is only in our analysis. Urine sample sent in boric acid to the laboratory. Microbiologists at the laboratory were blinded as to grouping. Final culture reports were classified as no growth, mixed, or pure. Mixed was defined as at least 2 organisms, and in most cases, specific identification of those organisms was not made. Those that were pure were further categorized according to species and colony-forming units per milliliter using a standard technique. Coliform organisms of 102 colonyforming units per milliliter or more were considered significant.

| | |
|---|---|
| Is the reference standard likely to correctly classify the target condition? | Yes |
| Were the reference standard results interpreted without knowledge of the results of the index test? | Yes |

**Could the reference standard, its conduct, or its interpretation have introduced bias?** **Risk of bias:** Low

**B. APPLICABILITY:**

**Is there concern that the target condition as defined by the reference standard does not match the review question?** **Concerns regarding applicability:** Low

# QUADAS-2

| ID: | 6 | Author: | Lifshitz | Year: | 2000 | Reviewer: | Anne |
|---|---|---|---|---|---|---|---|

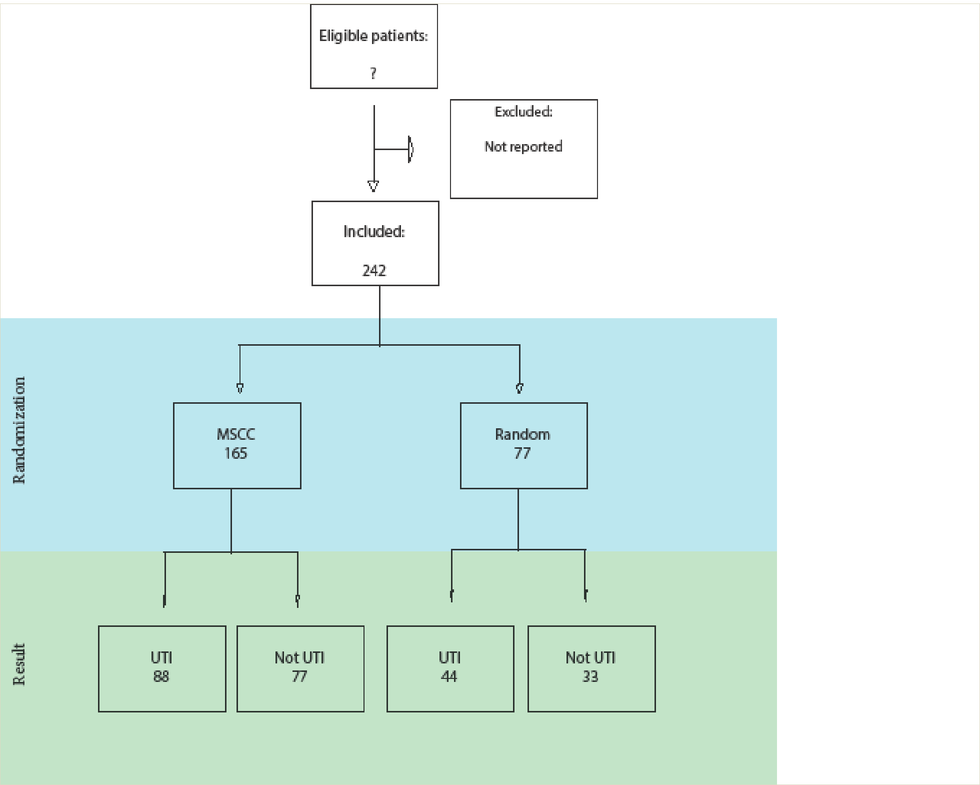


## DOMAIN 4: FLOW AND TIMING

### A. RISK OF BIAS

**Describe any patients who did not receive the index test(s) and or reference standard or who were excluded from the 2x2 table (refer to flow diagram):**

Not described who were excluded befor enrollment. Otherwice described.

**Describe the time interval and any interventions between index test(s) and reference standard:**

This study is not investigating paired samples but is a RCT and cannot perform well in this domain. The risk of bias is naturally high when the randomized setting is used to investigate a diagnostic test since we cannot conclude the performance of the test for individual patients.

| | |
|---|---|
| Was there an appropriate interval between index test and reference standard? | No |
| Did all patients receive a reference standard? | No |
| Did patients receive the same reference standard? | No |
| Were all patients included in the analysis? | Yes |

**Could the patient flow have introduced bias?** High

# QUADAS-2

| ID: | 7 | Author: | Bradbury | Year: | 1988 | Reviewer: | Anne |
|---|---|---|---|---|---|---|---|

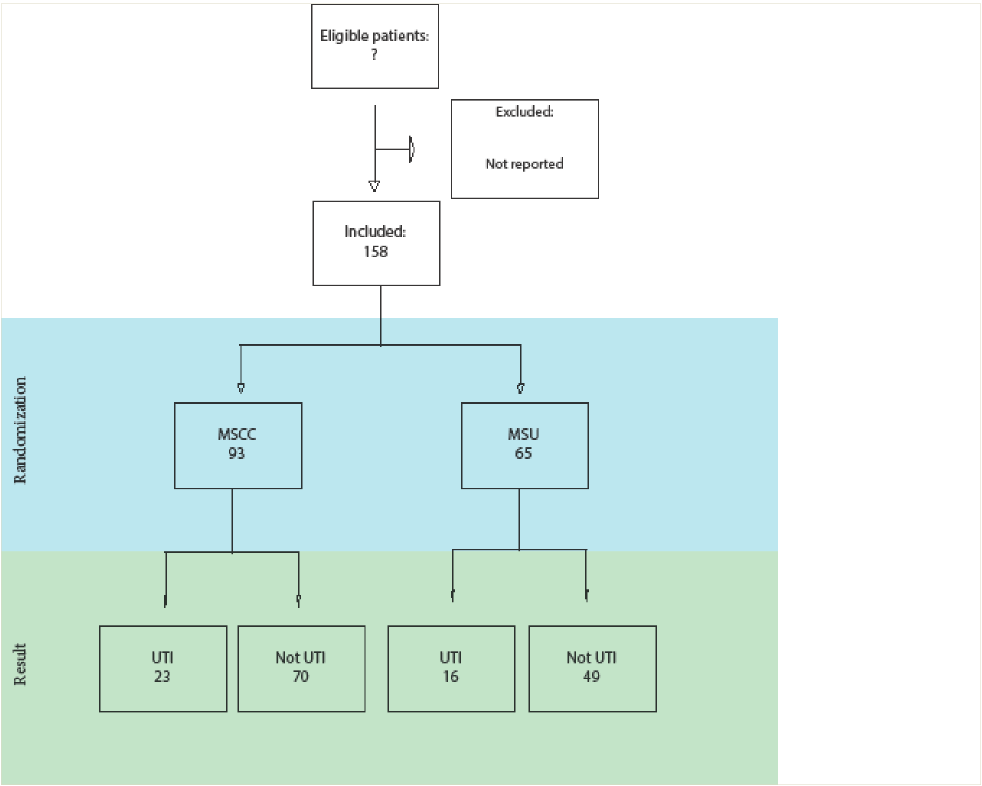


## DOMAIN 1: PATIENT SELECTION

### A. RISK OF BIAS

**Describe methods of patient selection:**

The study was carried out in a five-doctor practice with a popula- tion of around 11 000. A total of 316 urine specimens were collected from 158 female patients with suspected urinary tract infection. Exclusion criteria relevant.

| | |
|---|---|
| Was a consecutive or random sample of patients enrolled? | Yes |
| Was a case-control design avoided? | Yes |
| Did the study avoid inappropriate exclusions?: | Yes |

**Could the selection of patients have introduced bias?** **Risk of bias:** Low

### B. APPLICABILITY:

**Describe included patients:**

Adult symptomatic women presenting in primary care.

**Do the included patients and setting match the question?** **Concerns regarding applicability:** Low

# QUADAS-2

| ID: | 7 | Author: | Bradbury | Year: | 1988 | Reviewer: | Anne |
|---|---|---|---|---|---|---|---|

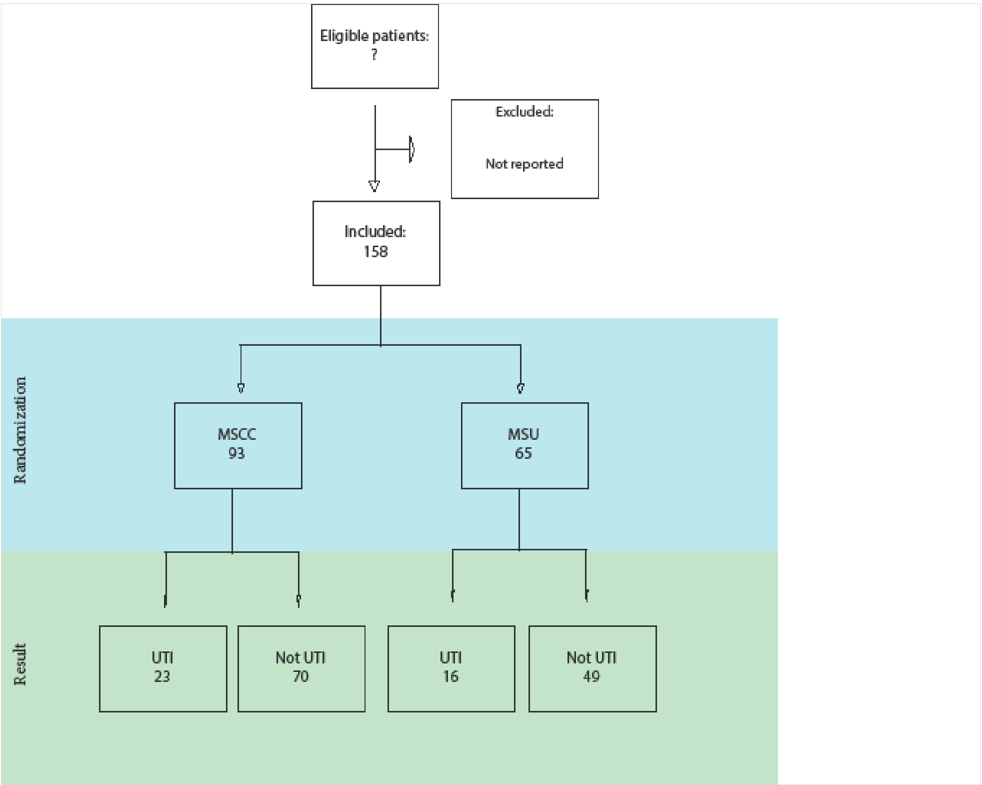


## DOMAIN 2: INDEX TEST

**A. RISK OF BIAS**

**Describe the index test and how it was conducted and interpreted**

Mid-stream urine without prior cleasning. In the laboratory the specimens were examined microscopically for the presence and number of white and red blood cells and the presence of casts. The criteria used to indicate the presence of infection were the number and culture-purity of the organisms isolated in the presence of significant numbers of white blood cells per litre of urine. A 'definite' infection was classified as one with greater than 10.000 cfu/ml of urine in the presence of significant numbers of white cells

| | |
|---|---|
| Were the index test results interpreted without knowledge of the results of the reference standard? | Yes |
| If a threshold was used, was it pre-specified? | Yes |

**Could the conduct or interpretation of the index test have introduced bias?** **Risk of bias:** Low

**B. APPLICABILITY:**

**Is there concern that the index test, its conduct, or interpretation differ fromt he review question** **Concerns regarding applicability:** Low

# QUADAS-2

| ID: | 7 | Author: | Bradbury | Year: | 1988 | Reviewer: | Anne |
|---|---|---|---|---|---|---|---|

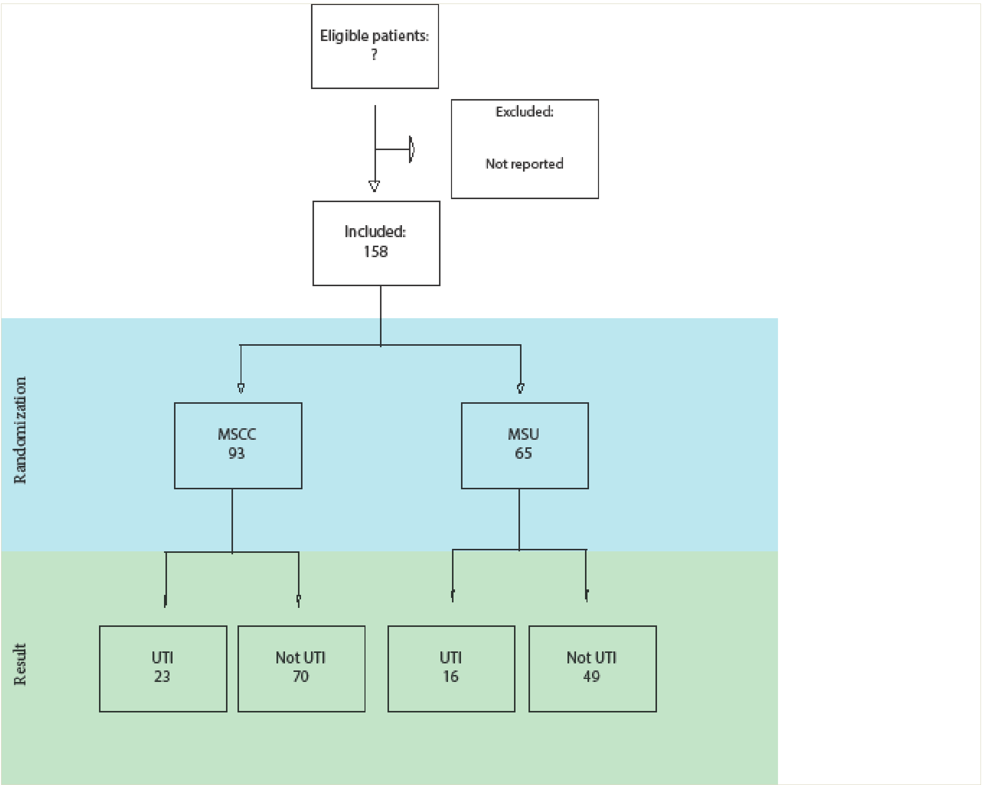


## DOMAIN 3: REFERENCE STANDARD

**A. RISK OF BIAS**

**Describe the reference standard and how it was conducted and interpreted::**

Mid-stream urine with prior cleasning with soap and water. In the laboratory the specimens were examined microscopically for the presence and number of white and red blood cells and the presence of casts. The criteria used to indicate the presence of infection were the number and culture-purity of the organisms isolated in the presence of significant numbers of white blood cells per litre of urine. A 'definite' infection was classified as one with greater than 10.000 cfu/ml of urine in the presence of significant numbers of white cells

| | |
|---|---|
| Is the reference standard likely to correctly classify the target condition? | Yes |
| Were the reference standard results interpreted without knowledge of the results of the index test? | Yes |

**Could the reference standard, its conduct, or its interpretation have introduced bias?** **Risk of bias:** Low

**B. APPLICABILITY:**

**Is there concern that the target condition as defined by the reference standard does not match the review question?** **Concerns regarding applicability:** Low

# QUADAS-2

| ID: | 7 | Author: | Bradbury | Year: | 1988 | Reviewer: | Anne |
|---|---|---|---|---|---|---|---|

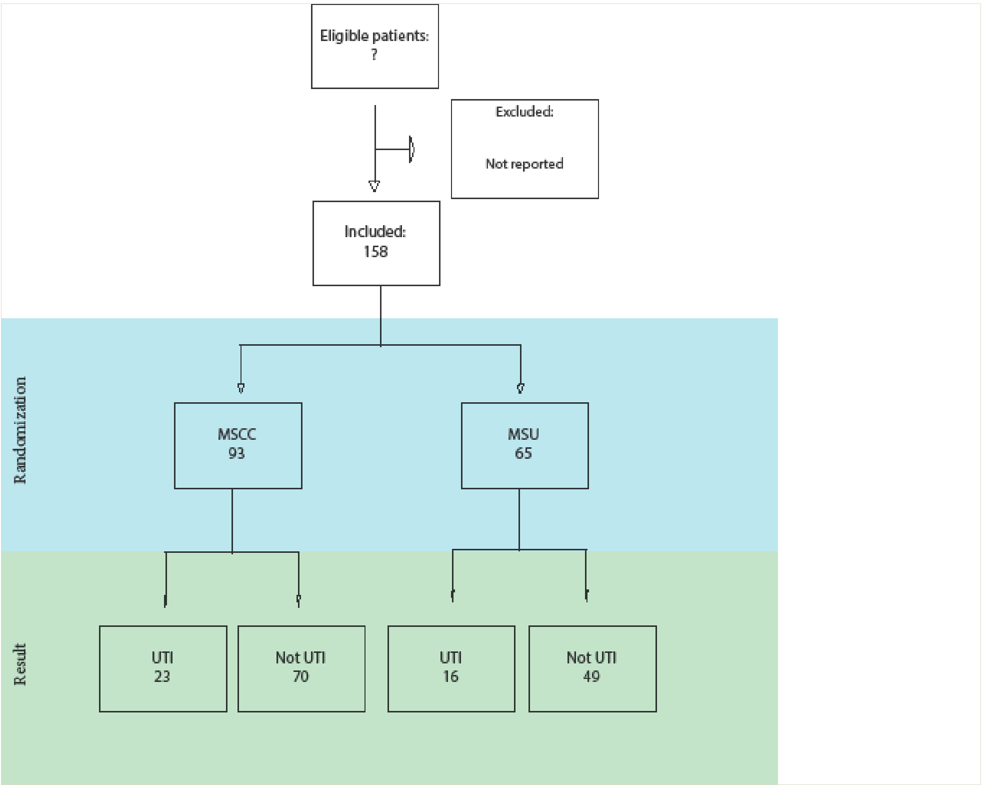


## DOMAIN 4: FLOW AND TIMING

### A. RISK OF BIAS

**Describe any patients who did not receive the index test(s) and or reference standard or who were excluded from the 2x2 table (refer to flow diagram):**

Not describing exclusions prior to randomization but otherwice described

**Describe the time interval and any interventions between index test(s) and reference standard:**

This study is not investigating paired samples but is a RCT and cannot perform well in this domain. The risk of bias is naturally high when the randomized setting is used to investigate a diagnostic test since we cannot conclude the performance of the test for individual patients.

| | |
|---|---|
| Was there an appropriate interval between index test and reference standard? | No |
| Did all patients receive a reference standard? | No |
| Did patients receive the same reference standard? | No |
| Were all patients included in the analysis? | Unclear |

**Could the patient flow have introduced bias?** High
